# Integrating causal human genetics and *In vivo* transcriptomics to uncover a shared lipid-centric architecture in metabolic and neurocognitive disease

**DOI:** 10.3389/fmolb.2025.1712198

**Published:** 2026-01-27

**Authors:** Yi Li, Zhu Ni, Xiao-Yong Xia, Na Cheng, Yu Bo, Junwen He, Yang He, Xiang-Yu Meng, Xu Wang, Xuan Xu

**Affiliations:** 1 School of Life Sciences, Anhui Medical University, Hefei, Anhui, China; 2 School of Biomedical Engineering, Anhui Medical University, Hefei, Anhui, China; 3 College of Informatics, Huazhong Agricultural University, Wuhan, Hubei, China; 4 Health Science Center, Medical School, Hubei Minzu University, Enshi, Hubei, China

**Keywords:** Alzheimer’s disease, lipid metabolism, machine learning, metabolic syndrome, systems genetics

## Abstract

**Background:**

Metabolic disorders and neurocognitive diseases frequently co-occur, yet the specific mechanisms driving this comorbidity remain elusive. While epidemiological associations are well-documented, the causal links between these conditions are complex and incompletely understood, necessitating a systems-level investigation into their shared biological architecture.

**Methods:**

This study integrates large-scale human genetics with experimental *in vivo* transcriptomics and computational chemistry to elucidate these shared pathways. Specifically, an AD-like murine model was used to experimentally prioritize a core network of 13 dysregulated genes within a pathologically relevant context.

**Results:**

Network-informed Mendelian randomization identified bidirectional causalities, including a 14% elevated dementia risk from type 2 diabetes and protective effects of obesity against parental Alzheimer’s disease (AD). The study identified a signature encompassing key lipid metabolism hubs *APOE, CLU*, and *LDLR*. This signature serves as a critical biological filter, anchoring human genetic associations by providing direct evidence of their dysregulation in a neurodegenerative environment. Subsequent chemical enrichment and molecular docking analyses indicated that these experimentally-prioritized targets are engaged by both therapeutic agents (e.g., valproic acid) and environmental toxins (e.g., benzo[a]pyrene).

**Conclusion:**

This multi-modal investigation provides a robust framework that converges on a high-confidence, 13-gene signature of lipid dysregulation as a central mechanistic interface, offering a powerful set of prioritized targets for future functional validation and therapeutic development at the metabolic-neurocognitive nexus.

## Introduction

Age-related disorders, particularly metabolic and neurocognitive disorder, represent an escalating global health challenge. Neurodegenerative conditions, with Alzheimer’s disease (AD) as the most prevalent form of dementia, place a profound burden on affected individuals and healthcare systems worldwide (Manly et al., 2022). In parallel, metabolic disorders such as type 2 diabetes (T2D), obesity, and hypertension have reached epidemic proportions (Klein et al., 2022; Pasdar et al., 2024). Epidemiological studies consistently demonstrate strong associations between these disease classes; for example, midlife obesity and T2D are recognized risk factors for late-life dementia (Marrano et al., 2023; Toledano et al., 2024). However, observational findings are inherently susceptible to confounding and reverse causation, leaving the biological mechanisms and directionality of these relationships incompletely understood (Ezkurdia et al., 2023; Weijie et al., 2024).

Previous efforts to clarify these links have often relied on genetic approaches that use inherited variants as proxies for modifiable exposures, enabling causal inference with reduced confounding (Zhang et al., 2021; Hong et al., 2025; Wang F. et al., 2025). These studies have provided important insights, suggesting that metabolic traits may influence neurodegenerative risk (Domenighetti et al., 2024; Guo et al., 2025). However, while bidirectional MR has begun to illuminate these reciprocal influences, such analyses have largely been restricted to isolated, pairwise assessments. Consequently, a systems-level map that models the complex interplay and shared genetic architecture across a network of metabolically-linked neurocognitive disorder remains a critical, unaddressed gap. Moreover, translating statistical associations into actionable biological insight requires integration with functional genomics data to illuminate the molecular processes that mediate these effects (Ebrahim and Davey Smith, 2008; Tyner, 2017).

Here, we present a multi-stage analytical framework designed to map the shared causal architecture between metabolic and neurocognitive disease and to identify biologically meaningful molecular drivers. Critically, to avoid circular reasoning and selection bias, our pipeline begins with hypothesis-free, genome-wide inputs for both genetic (GWAS) and transcriptomic datasets. Rather than funneling data through pre-selected domains, we allowed key pathways to emerge naturally from the intersection of independent data modalities. We first apply a network-guided causal inference strategy to uncover robust, biologically coherent trait–trait relationships and cluster them into functional modules, thereby enhancing both statistical power and interpretability. Genetic variants supporting these relationships are then mapped to putative effector genes. To experimentally prioritize these candidates and validate their dysregulation in a relevant pathological context, we established an AD-like murine model and performed hippocampal RNA-sequencing. This approach enabled us to filter and rank genetically-implicated candidates based on their actual expression changes in a disease-relevant tissue environment, thereby bridging statistical associations with biological plausibility. The resulting set of high-confidence, dysregulated genes is then explored for therapeutic and environmental relevance by mapping their interactions with chemicals and drugs using the Comparative Toxicogenomics Database (CTD), followed by molecular docking simulations. These computational analyses are employed not as definitive validation, but to explore the structural plausibility of binding and to generate mechanistic hypotheses regarding how prioritized environmental agents might structurally engage with the identified gene targets.

**FIGURE 1:**
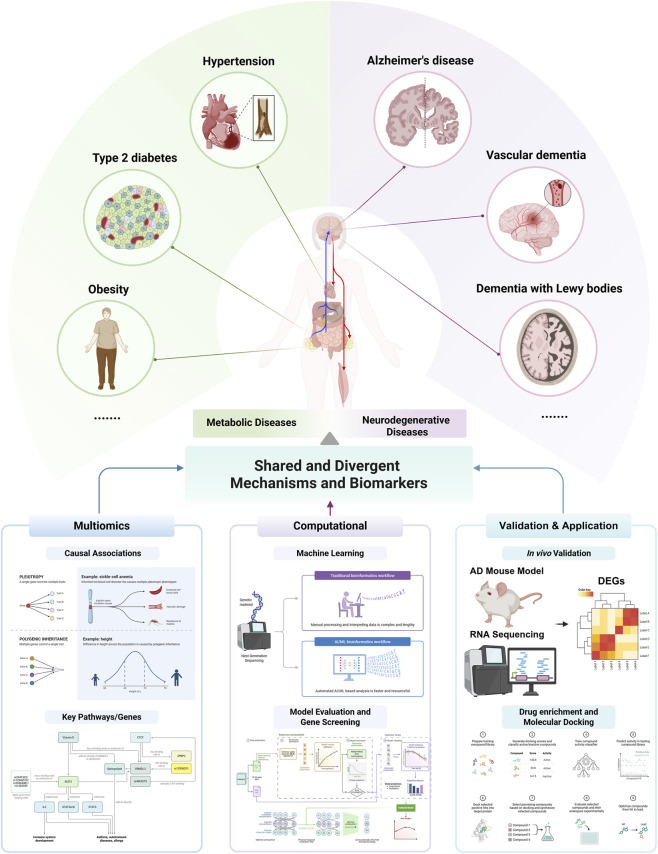
Schematic overview of the multi-stage analytical framework for dissecting the shared causal architecture between metabolic and Neurocognitive Disorder. The analytical pipeline begins with a network-informed, bidirectional Mendelian randomization (MR) analysis to establish robust causal relationships between a curated panel of metabolic and neurodegenerative traits. Significant causal pairs are organized into functional modules. Genetic instruments (SNPs) underlying these causal links are mapped to their putative effector genes. To experimentally validate and prioritize these candidates within a relevant pathological context, we established an AD-like murine model and performed hippocampal RNA-sequencing, generating a high-confidence set of dysregulated genes. In a parallel stream, the biological pathways enriched by the initial set of putative genes are evaluated for functional relevance using a pathway-centric machine learning framework on six disease-specific transcriptomic datasets. In the final stage, the experimentally-prioritized driver genes from our *in vivo* model are queried against the Comparative Toxicogenomics Database (CTD) to identify potential interactions with drugs and environmental chemicals. High-priority chemical-gene pairs are then subjected to molecular docking analysis to provide structural evidence supporting these interactions. Overall, this integrated strategy is designed to systematically progress from broad genetic causal inference, through experimental validation in a disease-relevant model, to specific mechanistic insights and the identification of therapeutically relevant molecular targets.

We hypothesize that anchoring large-scale genetic causal inference with experimental transcriptomic data from an *in vivo* model will provide a robust framework for identifying a core set of shared molecular pathways underlying the comorbidity of metabolic and neurodegenerative disorders. By bridging causal inference with experimental prioritization and mechanistic insight, this study aims to (1) construct a systems-level causal landscape connecting metabolic and neurocognitive disorder, with a primary focus on identifying and dissecting bidirectional relationships that suggest shared feedback mechanisms; (2) experimentally prioritize and characterize the tissue-specific predictive utility of the key genes and pathways mediating these relationships within an *in vivo* pathological context; and (3) highlight molecular nodes that represent promising candidates for diagnostic, therapeutic, and preventive interventions, pending further functional validation studies. A schematic of the analytical strategy is provided in Figure 1 to illustrate the overall workflow.

## Methods

### Phenotype definitions and GWAS sources

To investigate the shared genetic architecture and potential causal pathways connecting metabolic disorders and neurocognitive disorder, we curated a focused panel of phenotypes representing two major categories of age-related disorders. The first category encompassed traits indicative of neurodegeneration and cognitive impairment, including AD and all-cause Dementia. The second category comprised classical metabolic disorders, namely hypertension, obesity, T2D, and non-alcoholic fatty liver disease (NAFLD), all of which have been implicated in the etiology and progression of cognitive decline.

Genome-wide association study (GWAS) datasets were retrieved from publicly accessible repositories, including the IEU OpenGWAS project (https://gwas.mrcieu.ac.uk/), the NHGRI-EBI GWAS Catalog (https://www.ebi.ac.uk/gwas/), and the FinnGen consortium (https://www.finngen.fi/en/consortium), ensuring standardized data processing and consistent quality control across studies. Neurodegenerative phenotypes were defined using both algorithm-derived endpoints from the UK Biobank (UKB, https://www.ukbiobank.ac.uk/) and registry-based clinical outcomes from FinnGen. The UKB-derived trait *ieu-b-5067* captured AD status based on a composite definition incorporating hospital admission records, cognitive performance scores, and death registry data, analyzed using the BOLT-LMM framework (Mitchell et al., 2019). From FinnGen, we included three distinct AD endpoints—*finn-b-G6_AD_WIDE* (broad definition), *finn-b-AD_LO* (late-onset AD), and *finn-b-AD_AM* (atypical or mixed AD)—as well as two dementia-related phenotypes, *finn-b-KRA_PSY_DEMENTIA* (general dementia) and *finn-b-F5_ALZHDEMENT* (Alzheimer-related dementia).

Metabolic phenotypes were selected for their relevance to systemic vascular and metabolic dysfunction. Hypertension was represented by *ukb-b-12493*, corresponding to ICD-10 code I10 (essential hypertension), and *ieu-b-5144*, which defined early-onset hypertension based on internal UKB criteria. Obesity phenotypes included *ieu-a-1096* (childhood obesity, EGG consortium) and *ebi-a-GCST001475* (adult obesity, GIANT consortium), both derived from the meta-analysis by Bradfield et al. (2012). T2D was represented by four independent datasets: two from the Million Veteran Program (MVP)—*ebi-a-GCST90013892* and *ebi-a-GCST90013942*, analyzed using Firth and saddlepoint approximation (SPA) correction models, respectively (Mbatchou et al., 2021)—and two BMI-adjusted trans-ancestry datasets, *ebi-a-GCST007518* and *ebi-a-GCST007516*, from the meta-analysis by Mahajan et al. (2018). To capture hepatic metabolic contributions, two NAFLD datasets were included: *ebi-a-GCST90091033* (Ghodsian et al., 2021) (histologically confirmed liver fat accumulation) and *ebi-a-GCST90054782* (imaging-based liver fat content) (Ghodsian et al., 2021). These phenotypes provide important links between adiposity, hepatic lipid metabolism, and systemic inflammation, processes increasingly implicated in neurodegenerative pathology.

This ontology-driven phenotype selection established a robust foundation for investigating potential bidirectional genetic relationships between metabolic dysfunction and neurocognitive decline. Detailed phenotype definitions, sample sizes, ancestry compositions, and SNP coverage are provided in Table 1 and Supplementary Table S1.

**TABLE 1:** Characteristics of GWAS datasets.

Trait ID	Trait description	Sample size	PMID	Year	Population	nsnp
ieu-b-5067	Alzheimer’s disease (UKB, algorithm-defined)	488,285	35564004	2022	European	12,321,875
finn-b-G6_AD_WIDE	Alzheimer’s disease, wide definition (FinnGen)	—	34594039	2021	European	16,380,462
finn-b-AD_LO	Alzheimer’s disease (late onset)	∼2,670 cases	34594039	2021	European	214,871
finn-b-AD_AM	Alzheimer’s disease (atypical/mixed)	∼800 cases	34594039	2021	European	214,893
finn-b-KRA_PSY_DEMENTIA	All-cause Dementia	∼5,900 cases	34594039	2021	European	212,859
finn-b-F5_ALZHDEMENT	Dementia in Alzheimer’s disease	∼2,200 cases	34594039	2021	European	209,487
ukb-b-12493	Essential hypertension (ICD10: I10)	463,010	29844963	2018	European	9,851,867
ieu-b-5144	Early-onset hypertension (UKB)	462,826	—	2024	European	12,321,875
ieu-a-1096	Childhood obesity	13,848	22484627	2012	European	2,442,739
ebi-a-GCST001475	Obesity (adult)	13,848	22484627	2012	European	2,430,514
ebi-a-GCST90013892	Type 2 diabetes (Firth correction)	406,831	34017140	2021	European	11,039,026
ebi-a-GCST90013942	Type 2 diabetes (SPA correction)	406,831	34017140	2021	European	11,038,957
ebi-a-GCST007518	T2D (BMI-adjusted)	298,957	29632382	2018	European	190,208
ebi-a-GCST007516	T2D (BMI-adjusted)	298,957	29632382	2018	European	190,208

### Bidirectional two-sample Mendelian Randomization analysis

Following the curation of GWAS datasets, we conducted a bidirectional two-sample analysis to systematically evaluate the causal relationships between the selected metabolic and neurodegenerative traits. This bidirectional framework was applied in two complementary directions: in the forward analysis, metabolic disorders were treated as exposures and neurodegenerative traits as outcomes; conversely, in the reverse analysis, neurodegenerative traits served as exposures and metabolic disorders as outcomes. To reduce potential confounding from population stratification, all analyses were restricted to individuals of European ancestry.

For each exposure trait, we identified independent genetic instruments by selecting single nucleotide polymorphisms (SNPs) achieving genome-wide significance (*P* < 5 × 10^−8^) and applying linkage disequilibrium (LD) clumping (*r* ^2^ < 0.001 within a 1,000 kb window). Effect estimates for these instruments were then extracted from the outcome GWAS datasets. To ensure valid causal estimates, allele harmonization was performed to align effect alleles between exposure and outcome datasets, resolve strand ambiguity for palindromic SNPs, and remove instruments that could not be reliably matched.

The primary causal effects were estimated using the random-effects inverse-variance weighted (IVW) method. Robustness was evaluated using several sensitivity analyses: MR-Egger regression to test for directional pleiotropy (a non-significant intercept, *P* > 0.05, indicating absence of bias), Cochran’s Q statistic to quantify heterogeneity among SNP-specific estimates, leave-one-out analysis to detect disproportionately influential instruments, and Radial MR (both IVW- and MR-Egger-weighted) to identify and down-weight outlier SNPs.

A causal association was considered robust and reported as a qualified finding only if it met three stringent criteria: (1) a statistically significant effect in the IVW analysis (*P* < 0.05); (2) no evidence of directional pleiotropy (MR-Egger intercept *P* > 0.05); and (3) no significant heterogeneity across instruments (Cochran’s Q test *P* > 0.05). To ensure the validity of the causal direction and address the potential for bias in highly pleiotropic regions, we rigorously applied Steiger filtering (Hemani et al., 2017). This procedure calculates the variance explained in the exposure *versus* the outcome, removing instruments that explain more variance in the outcome to mitigate reverse causation. Furthermore, regarding the *APOE* locus (chr19:44.9–45.9 Mb), while we acknowledge its extensive pleiotropy, we elected to retain instruments within this region to capture the complete genetic architecture shared between lipid metabolism and neurodegeneration (Kunkle et al., 2019). The application of Steiger filtering ensures that these powerful genetic signals are statistically anchored to the hypothesized causal direction rather than being artifacts of reverse causality. Odds ratios (ORs) and 95% confidence intervals (CIs) were derived from the beta coefficients for all qualified associations. All statistical analyses were performed in R using the “TwoSampleMR” (Hemani et al., 2018) and “RadialMR” (Bowden et al., 2018) packages.

### Network-guided integration and filtering

To integrate the results systematically, we collated MR outputs from multiple independent analyses, harmonizing them into two master tables representing forward and reverse directions. Each record was assigned a unique identifier reflecting its original source. Using the forward-direction results, we constructed a trait-level causal network, in which nodes represented traits (exposures or outcomes) and edges represented statistically significant causal links. The Walktrap community detection algorithm was applied to partition this network into densely connected modules, capturing groups of biologically or functionally related traits.

We then applied a stringent network-informed filtering strategy to prioritize high-confidence causal relationships. Specifically, we defined a set of “core network relationships,” representing exposure–outcome pairs in which both traits resided within the same module, reasoning that intra-modular relationships were more likely to reflect coherent biological mechanisms. This curated core set was used as a bidirectional filter: in the forward analysis, only pairs matching the core set were retained, whereas in the reverse analysis, only those pairs exactly inverting a core forward relationship (i.e., exposure and outcome reversed) were retained. The resulting dataset comprised bidirectionally evaluated, module-consistent relationships. Each entry was annotated with its network module identifier and subsequently used for downstream analyses.

### Identification, prioritization, and functional annotation of key genetic instruments

To determine the genetic variants underlying the observed causal relationships, we applied a multi-stage process encompassing SNP filtering, prioritization, and functional annotation. In the initial step, instrumental SNPs were selected using a stringent network-guided criterion derived from the previously defined core network relationships. These core relationships represent exposure–outcome pairs in which both traits reside within the same computationally defined biological module. For forward-direction analyses, a SNP was retained only if its exposure–outcome pair matched a core network relationship. For reverse-direction analyses, the selection criterion was symmetrically applied, retaining SNPs only when their exposure–outcome pair precisely inverted a core forward relationship. This approach ensured that only SNPs linked to robust, module-supported causal pathways were carried forward for subsequent analyses.

The retained SNPs were then prioritized based on their frequency of association, calculated as the number of distinct module-supported causal relationships in which each SNP served as an instrument. This frequency-based prioritization was performed separately for each original data source and for each causal direction (forward and reverse), enabling identification of robust instruments repeatedly implicated across multiple independent causal tests.

The final analytical step involved functional annotation of the prioritized SNPs to identify candidate effector genes. Positional mapping was performed using the human genome build hg19 as a reference. A candidate gene was assigned to a SNP if it was the closest annotated gene located within a 50 kb flanking region upstream or downstream of the SNP’s genomic coordinates. These functionally annotated SNP–gene pairs provided the foundation for subsequent biological pathway and network analyses.

### Pathway and ontology enrichment analysis of key genes

To investigate the collective biological functions of the prioritized genes, we performed formal gene set enrichment analysis (GSEA) to identify biological pathways and ontology terms significantly over-represented within our gene lists. The reference for this analysis was the C5 ontology gene sets collection from the Molecular Signatures Database (Liberzon, 2014; Castanza et al., 2023) (MSigDB v2025.1. Hs, https://www.gsea-msigdb.org/gsea/msigdb). This resource integrates curated gene sets from two primary ontology sources: the Gene Ontology (GO) project, covering its three core domains—Biological Process (BP), Cellular Component (CC), and Molecular Function (MF)—and the Human Phenotype Ontology (HPO). To ensure functional relevance, the MSigDB C5 collection excludes overly broad gene sets (more than 2,000 members) and overly specific sets (fewer than five members). The analytical background was defined as the complete set of unique genes represented across all pathways in this collection.

The enrichment analysis was conducted separately for each group defined by a unique combination of original GWAS data source and direction of causal analysis (forward or reverse). For each group, we compiled a query list of unique key genes identified in the preceding SNP prioritization step. The statistical significance of enrichment for each gene set was evaluated using a one-sided hypergeometric test, which measures whether the observed overlap between the query genes and a given pathway exceeds the overlap expected by random chance. To correct for multiple testing across thousands of pathways, raw *P*-values from the hypergeometric tests were adjusted using the Benjamini–Hochberg False Discovery Rate (FDR) procedure. Gene sets were considered significantly enriched if they met the threshold of an FDR-adjusted *P* < 0.05.

### Transcriptomic data acquisition and differential expression analysis

To characterize transcriptional alterations associated with disease states, we obtained six publicly available transcriptomic datasets from the Gene Expression Omnibus (GEO, https://www.ncbi.nlm.nih.gov/geo/): GSE5281 (AD) (Readhead et al., 2018), GSE140830 (all-cause Dementia), GSE122063 (VaD) (McKay et al., 2019), GSE184050 (T2D) (Chen et al., 2022), GSE262828 (Hypertension) (Ananthamohan et al., 2024), and GSE77962 (obesity) (Tareen et al., 2020). Each dataset provided pre-processed gene expression matrices and metadata specifying sample diagnosis and clinical status. Detailed dataset information, including sample size, tissue origin, and experimental platform, is provided in Table 2 and Supplementary Table S2.

**TABLE 2:** Characteristics of gene expression datasets.

Disease	GEO NO.	Sample size	Description	PMID
AD	GSE5281	Total: 161 Controls: 74 Cases: 87 (cortical layer III neurons)	This study profiled gene expression to identify molecular signatures of AD and aging. To eliminate tissue heterogeneity, laser capture microscopy (LCM) was used to isolate layer III pyramidal cells from six specific brain regions (entorhinal cortex, hippocampus, medial temporal gyrus, posterior cingulate, superior frontal gyrus, and primary visual cortex). Gene expression was measured using Affymetrix U133 plus 2.0 arrays.	17077275; 18332434; 29937276; 18270320
Dementia	GSE112681	Total: 542 Controls: 281 Cases: 261 (bvFTD: 80, PSP: 54, nfvPPA: 47, svPPA: 44, CBS: 36)	This study investigates the role of peripheral inflammation in dementia by analyzing gene expression profiles from total RNA obtained from peripheral blood. It compares a diverse set of dementia patients—including multiple Frontotemporal Dementia (FTD) spectrum disorders—to healthy controls to identify transcriptional signatures of inflammation.	29939990; 31118040
VaD	GSE131282	Total: 136 Controls: 44 Cases: 92 (Alzheimer’s disease: 56, vascular dementia: 36)	This study profiled gene expression on post-mortem frontal and temporal cortex tissue, comparing vascular Dementia (VaD), Alzheimer’s Disease (AD), and non-demented controls. VaD cases were selected for low Braak staging to minimize AD comorbidity, while AD and control cases were confirmed to have no infarcts. Gene expression was measured using Agilent human 8 × 60k v2 microarrays.c	30990880
Hypertension	GSE262828	Total: 89 Low blood pressure: 43 Mid/Elevated blood pressure: 13 High blood pressure: 33^```^	This study aims to identify molecular mechanisms of target organ Damage (TOD) in youth with primary hypertension. Using high-throughput sequencing (RNA-seq), it profiled gene expression in circulating peripheral blood mononuclear cells (PBMCs) from youth stratified by blood pressure levels and the presence of TOD. The study is part of a larger multi-omics investigation to define circulatory regulators involved in blood pressure-mediated TOD.	38948714
Obesity	GSE77962	Total: 152 (from 53 individuals) Baseline: ∼51 (inferred) After weight loss period: 50 After weight stable period: 51	This randomized controlled trial investigated the effect of weight loss rate on the adipose tissue transcriptome. Overweight and obese individuals were assigned to either a low-calorie diet (LCD) or a very-low-calorie diet (VLCD) to achieve similar total weight loss. Subcutaneous adipose tissue biopsies were collected at baseline, after the weight loss period, and after a subsequent weight stabilization period, with gene expression analyzed by microarray.c	30380678; 27840413; 32015415
T2D	GSE28894	Total: 116 Controls: 66 Cases: 50	This longitudinal study investigated gene expression changes in whole blood to identify transcriptional signatures associated with the progression to type 2 Diabetes (T2D). Using high-throughput sequencing (RNA-seq), it compared subjects who transitioned to T2D over time against controls who did not. A key gene network associated with T2D transition status was identified and subsequently validated.	35157052

To ensure consistency across datasets, a standardized analysis workflow was applied. Importantly, to avoid artifacts arising from tissue heterogeneity and batch effects, we did not merge the raw expression matrices. Instead, each dataset underwent independent normalization and differential expression (DE) analysis specific to its tissue of origin (brain, blood, or adipose). This strategy ensures that the identified gene signatures reflect robust disease-associated changes within each specific biological context, rather than technical disparities between tissue types. Samples were classified into two groups based on study metadata: a “disease” group representing the pathological condition of interest and a “healthy control” (HC) group. Differential gene expression (DGE) analysis was performed using the “limma” (Ritchie et al., 2015) package in R, suitable for both microarray and RNA sequencing (RNA-seq) data. For each gene, a linear model was fitted to evaluate expression differences between disease and control groups using a design matrix constructed to capture group effects. This strategy ensures that the identified gene signatures reflect robust disease-associated changes within each specific biological context, distinguishing shared pathological mechanisms (e.g., lipid dysregulation) from generic systemic inflammation.

To enhance statistical power and stabilize variance estimates, particularly in datasets with modest sample sizes, empirical Bayes moderation was applied to model coefficients using the eBayes function. This procedure moderates standard errors by borrowing information across all genes, providing robust estimates of differential expression.

For each gene, the analysis generated log_2_ fold-change (logFC) values to quantify the magnitude of expression differences, along with associated *P*-values and Benjamini–Hochberg false discovery rate (FDR)–adjusted *P*-values. These results provided both the effect size and statistical significance for transcriptional changes between disease and control cohorts.

### Pathway-centric machine learning framework

To build predictive models and identify key driver genes from the complex transcriptional landscapes of multiple disease states, we utilized six publicly available transcriptomic datasets covering both neurodegenerative conditions (AD, all-cause Dementia, and VaD) and metabolic disorders (T2D, Hypertension, and Obesity). From the biologically relevant gene sets identified through prior functional enrichment analyses, we selected subsets specifically associated with each disease category. Specifically, for each disease, the initial feature space for modeling was defined as the union of all genes belonging to pathways that were significantly enriched (FDR < 0.05) in the corresponding genetic analysis. These curated gene subsets served as the feature space for model construction, enabling the assessment of their predictive value for disease risk and the identification of potential molecular drivers.

Prior to model training, we applied a standardized preprocessing and feature selection pipeline to optimize the input data for each gene set. This pipeline consisted of three sequential steps: (1) Standardization, where gene expression values were transformed using a Z-score to ensure all features were on a comparable scale; (2) Low-Variance Filtering, to remove genes with near-constant expression that offer little discriminatory information; and (3) Univariate Feature Selection, which employed an ANOVA F-test to select the most significant genes associated with the disease status, dynamically adjusting the number of features to ensure a focused yet sufficiently complex feature set for modeling. This rigorous feature selection step was critical to mitigate the curse of dimensionality and reduce the risk of overfitting by focusing the models only on the most informative, pathway-restricted molecular features. To create a focused yet informative feature set, only genes with an ANOVA F-test *P*-value < 0.01 were retained for model training. This stringent, data-driven criterion ensures that only features with a strong univariate association with the phenotype are included, thereby reducing model complexity and the risk of including noisy variables.

### Machine learning model repertoire

To robustly assess the predictive potential of our selected gene features, we employed a diverse suite of machine learning algorithms. This multi-model strategy was chosen to mitigate the risk of model-specific bias and to systematically explore a wide range of underlying data structures, from simple linear relationships to complex, non-linear interactions. The repertoire included.

- Logistic Regression–a linear model providing a robust, interpretable performance baseline to establish benchmark performance.
- Support Vector Machine (SVM) and K-Nearest Neighbors (KNN) – more complex non-linear models capable of capturing intricate decision boundaries and non-parametric relationships, respectively.
- A variety of powerful ensemble methods, known for their high accuracy and inherent robustness against overfitting by aggregating predictions from multiple base learners. This included Random Forest and Extra Trees, which are parallel tree-based ensembles, as well as Gradient Boosting, XGBoost, and CatBoost, which are sequential ensembles that iteratively improve predictions.
- Finally, to test the capacity to capture high-order feature interactions that might be missed by traditional algorithms, we included state-of-the-art deep learning models specifically designed for tabular data. These were TabNet, which uses sequential attention to enhance interpretability, and FT-Transformer (Fourier Transform Transformer). While computationally intensive, the inclusion of models like FT-Transformer was justified by their potential to uncover novel predictive patterns in complex transcriptomic data, even with modest sample sizes, when combined with our rigorous cross-validation framework.

### Model evaluation and gene prioritization

A rigorous framework was implemented to evaluate model performance and identify key driver genes. All models were assessed using a 10-fold stratified cross-validation strategy to provide a robust and unbiased estimation of model performance on unseen data and to explicitly mitigate the risk of overfitting. During this process, we carefully monitored for significant discrepancies between training and validation performance metrics, which would indicate potential overfitting. The consistent performance observed across folds confirmed the generalizability of our models. Model performance was quantified using a comprehensive suite of metrics, including the area under the receiver operating characteristic curve (AUC-ROC), accuracy, F1-score, precision, recall, and Cohen’s kappa. For each pathway-specific model, a composite rank was derived by summing the individual performance ranks across all metrics, enabling the identification of models that consistently exhibited superior predictive performance.

To prioritize genes, we performed a hierarchical feature importance analysis restricted to the top five models as determined by the composite rank. Feature importance was extracted using methods appropriate to each model architecture: intrinsic importance scores were used for models with built-in ranking mechanisms (e.g., tree-based ensembles and linear models), whereas SHAP (SHapley Additive exPlanations) values were used for complex, non-linear models to estimate the marginal contribution of each gene to model predictions. The final comprehensive importance score for each gene was calculated by weighting its normalized importance from each of the top five models by the corresponding model’s composite rank, yielding a robust, performance-weighted measure of gene relevance within the diagnostic signature.

The machine learning model repertoire and evaluation framework were implemented using the following Python packages: pandas: v2.2.2, NumPy: v1.26.4, scikit-learn: v1.5.0, XGBoost: v2.1.0, CatBoost: v1.2.5, PyTorch: v2.3.0, pytorch_tabnet: v4.1.0, rtdl: v0.0.13, and SHAP: v0.45.0.

### Prioritization of key disease-associated genes

To derive a high-confidence list of key driver genes from the machine learning results, we implemented a multi-stage, data-driven prioritization pipeline. This approach integrated evidence from the independently trained models for each disease cohort. By comparing high-performing features across these distinct, tissue-specific models, we identified shared molecular drivers that transcend tissue boundaries. This result-level integration allows for the detection of convergent pathological mechanisms—such as lipid dysregulation—while filtering out tissue-specific noise or generic systemic inflammation markers that might dominate a merged dataset.

The process began by quantifying model reliability through a Composite Model Performance Score. For each model within each pathway-specific gene set, six performance metrics (accuracy, AUC, F1-score, precision, recall, and Cohen’s kappa) were normalized to a 0–1 scale and averaged to produce a single score representing the overall predictive utility of the model.

Next, we calculated a Performance-Weighted Importance Score for each gene by multiplying its raw feature importance (as computed in the machine learning pipeline) by the Composite Model Performance Score of the model that identified it. This ensured that gene importance was up-weighted when derived from a robust, high-performing model and down-weighted when originating from a lower-performing one.

A final Gene Prioritization Score was then calculated for each gene within each disease dataset by summing its Performance-Weighted Importance Scores across all relevant pathways and models. This aggregation rewarded genes that were consistently identified as important across multiple predictive contexts. Genes were ranked in descending order based on this score, and a stringent selection criterion was applied: only the top 1% of genes for each disease context were retained for downstream analysis, yielding a high-confidence list of key driver genes.

### Chemical-gene interaction network analysis

To explore the therapeutic potential of the prioritized genes, we interrogated the Comparative Toxicogenomics Database (CTD) (Wiegers et al., 2025) (http://ctdbase.org/), a curated resource detailing chemical–gene interactions and their relevance to human health. As of the July 2025 release, the CTD contained more than 3.1 million manually curated chemical–gene interactions, encompassing approximately 15,000 unique chemicals and 57,000 genes. This dataset is augmented by systematic integration with other authoritative bioinformatics resources, including NCBI Gene, KEGG, Reactome, GO, PubChem, ChemIDplus, and PubMed.

For each disease context, we identified the top five hub chemicals, defined as those interacting with the largest number of prioritized gene targets. These chemicals were classified as either “Drugs” or “Compounds” according to CTD annotations, enabling differentiation between potential therapeutic agents and environmental exposures. For each hub chemical, we constructed and analyzed chemical–gene interaction networks, representing chemicals and genes as nodes and curated interactions as edges. Each interaction was further annotated by its reported mode of action (e.g., activation, inhibition, or binding). To detect latent structural relationships, we applied the Louvain community detection algorithm, which identified densely interconnected modules of chemicals and genes.

Finally, to facilitate cross-disease comparisons, we integrated hub chemicals and their gene targets from all six disease contexts into a single bipartite network. This unified framework enabled the identification of shared therapeutic targets, potential pleiotropic effects, and molecular cross-talk among metabolic and neurodegenerative disorders.

### Animal model and behavioral assessment

All animal procedures were conducted in accordance with the guidelines and approved by the Institutional Review Board of Hubei Minzu University. Ten-week-old, specific-pathogen-free (SPF) male Kunming mice (n = 5 per group) were sourced from Sibeck Biotechnology (Henan, China). Following a 7-day acclimatization period, an oxidative stress-induced model of aging-associated cognitive impairment, which mimics key features of sporadic AD, was established. Unlike transgenic models that replicate familial amyloid pathology, the D-galactose/sodium nitrite (D-gal/NaNO2) regimen was selected to specifically induce systemic oxidative stress, mitochondrial dysfunction, and metabolic dysregulation—the core shared pathways identified in our computational analysis (Rehman et al., 2017). The model group received daily subcutaneous injections of D-galactose (120 mg/kg/day) and sodium nitrite (55 mg/kg/day) for 60 consecutive days. The control group received vehicle injections. Both D-galactose and sodium nitrite were purchased from Aladdin Biochemical Technology Co., Ltd. (Shanghai, China).

Cognitive function was evaluated using the Morris Water Maze (MWM) test. For the spatial learning acquisition phase, mice were trained for five consecutive days to locate a hidden platform, with the escape latency recorded in each trial. On the sixth day, a probe trial was conducted by removing the platform to assess memory retention. The frequency of platform crossings and the time spent in the target quadrant were quantified as primary measures of spatial memory. All animal experiments were conducted under the ethical guidelines of the Hubei Minzu University Institutional Review Board.

### Molecular analyses of hippocampal tissue

Following behavioral assessments, mice (n = 3 per group) were euthanized *via* intraperitoneal injection of sodium pentobarbital (100 mg/kg). Euthanasia was confirmed by the absence of corneal reflex, respiration, and heartbeat. Immediately following euthanasia, hippocampal tissues were rapidly dissected, flash-frozen in liquid nitrogen, and stored at −80 °C for subsequent molecular analysis (Meng et al., 2021).

Total RNA was extracted from the hippocampal tissue and subjected to global transcriptomic profiling. RNA sequencing libraries were prepared according to the manufacturer’s protocol and sequenced on an Illumina platform. The resulting raw sequencing data underwent a standard bioinformatic pipeline, which included quality control, alignment to the reference genome (e.g., GRCm39), and transcript quantification. Differential expression analysis was then performed to identify significant transcriptomic alterations between the model and control groups.

To validate key gene expression changes identified by RNA-seq, we quantified the relative mRNA levels of the neuronal synapse marker, *Grin2a*, and the microglial inflammation marker, *Tnfrsf11b*. Expression was normalized to the endogenous control gene, *Gapdh*. The specific primers used for amplification were as follows:

Grin2a:
  Forward: 5′-ACGTGACAGAACGCGAACTT-3′
  Reverse: 5′-TCAGTGCGGTTCATCAATAACG-3′
Tnfrsf11b:
  Forward: 5′-CCTTGCCCTGACCACTCTTAT-3′
  Reverse: 5′-CACACACTCGGTTGTGGGT-3′
Gapdh:
  Forward: 5′-GGTTGTCTCCTGCGACTTCA-3′
  Reverse: 5′-TGGTCCAGGGTTTCTTACTCC-3′

### Molecular docking analysis of prioritized chemical-target pairs

Molecular docking simulations were performed to predict binding affinities and characterize potential interaction modes between prioritized chemicals and their corresponding protein targets. Candidate chemical–protein pairs were selected based on curated interactions from the CTD, ensuring that each chemical was linked to at least one high-priority gene identified by the machine learning analysis and enriched across multiple disease contexts.

Three-dimensional protein structures were retrieved from the Protein Data Bank (PDB, https://www.rcsb.org/), and structural preparation was performed using the Molecular Operating Environment (MOE) software. Preprocessing steps included removal of co-crystallized water molecules and ligands, addition of hydrogen atoms, and assignment of protonation states of ionizable residues under physiological pH (7.4) using the Protonate 3D module. Canonical 2D ligand structures were obtained from the PubChem database, converted into 3D conformations, and energy-minimized using the AMBER10:EHT force field to generate stable geometries.

Docking was carried out using a rigid-receptor, flexible-ligand approach. Binding sites were identified using the Site Finder module, and ligand placement was performed using the Triangle Matcher algorithm, generating 30 distinct binding poses per ligand. These poses were scored using the London dG scoring function, which estimates the Gibbs free energy of binding (ΔG). For each chemical–target pair, the top-scoring pose representing the most favorable binding configuration was selected for detailed characterization, including non-covalent interactions such as hydrogen bonding, hydrophobic contacts, and π–π stacking, to elucidate the molecular basis of ligand binding.

### Statistical analysis

All statistical analyses were performed in R (v4.3.0) and Python (v3.10). Genome-wide association signals were filtered at *P* < 5 × 10^−8^. Differential expression analyses applied the limma package with empirical Bayes moderation and Benjamini–Hochberg false discovery rate (FDR) correction (*P* < 0.05). Machine learning models were evaluated using 10-fold stratified cross-validation, with model performance assessed by AUC-ROC, accuracy, precision, recall, F1-score, and Cohen’s kappa. Functional enrichment analyses used a one-sided hypergeometric test with FDR adjustment (*P* < 0.05). Functional enrichment analysis of prioritized genes employed a one-sided hypergeometric test with FDR adjustment (*P* < 0.05). Chemical–gene network analyses used the Louvain community detection algorithm to identify functionally coherent interaction modules. Molecular docking results were evaluated based on London dG binding scores, with lower ΔG values indicating higher binding affinity.

## Results

### Network-informed Mendelian randomization reveals complex causal architectures

**FIGURE 2:**
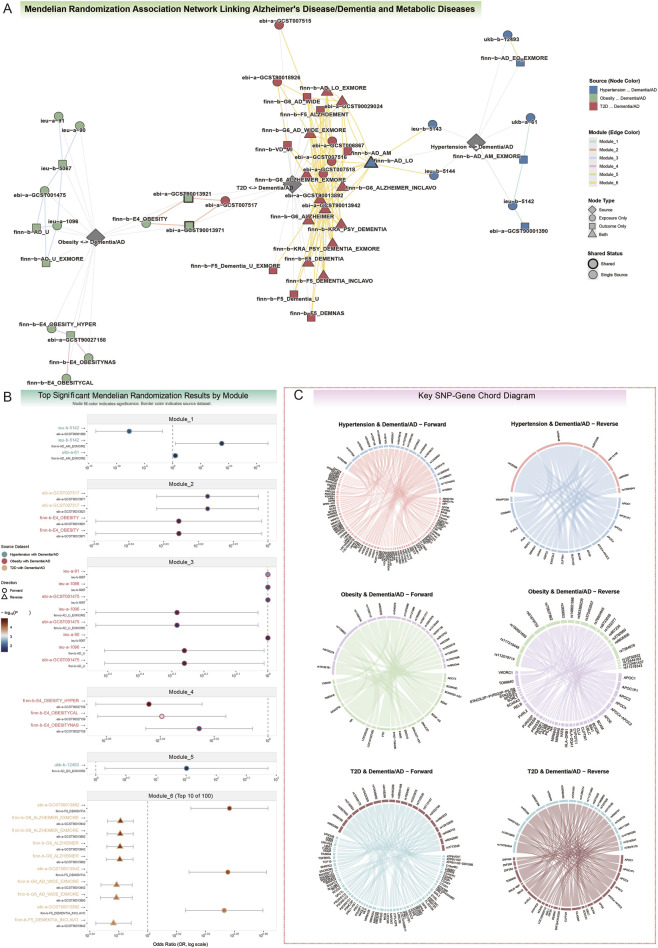
Mendelian randomization uncovers causal links and shared genetic architecture between metabolic and neurocognitive disorder. **(A)** The Mendelian Randomization association network systematically maps the causal architecture linking metabolic and Neurocognitive Disorder. The analysis partitions significant associations into six functionally distinct modules, indicated by edge color. This data-driven network reveals a complex landscape of both risk-conferring and protective effects. For instance, Module 1 highlights the contrasting effects of hypertension, which is protective against one dementia subtype while being a risk factor for another. Modules 2 and 4 are characterized by protective links, where obesity is associated with a reduced risk of AD. In contrast, Module 5 identifies a single, potent risk association between primary hypertension and early-onset AD. The largest and most interconnected cluster, Module 6, demonstrates significant bidirectional causality between Type 2 Diabetes (T2D) and dementia, indicative of an intricate feedback mechanism between these conditions. **(B)** Forest plots display the most significant Mendelian Randomization results, filtered to show up to the top 10 associations per module, ranked by p-value. For modules containing more than 10 significant findings, the label indicates that the top 10 are shown (e.g., Module 6, Top 10 of 100). The source metabolic disease category (Hypertension, T2D, or Obesity) for each association is indicated by the color of the exposure label on the y-axis. These plots powerfully illustrate key findings, such as the bidirectional relationship in Module 6, where forward analysis (T2D → Dementia) shows increased risk (OR > 1) and reverse analysis (Dementia → T2D) shows a protective effect (OR < 1). **(C)** Key SNP-Gene Chord Diagrams map the genetic instruments (SNPs) to their annotated genes for the primary causal associations. These diagrams reveal the genetic architecture mediating the interplay between metabolic disorders and AD/Dementia. • Hypertension and Dementia/AD: In the forward direction (Hypertension → Dementia/AD), instrumental variants map to genes including *ABHD16A*, *APOM*, *MTHFR*, and *NPPA.* In the reverse direction, the genetic instruments converge primarily on the *APOE/TOMM40* locus, with other implicated genes including *PVRL2* and *BCAM.* • Obesity and Dementia/AD: Forward analysis implicated well-established obesity-related loci, including *FTO*, *BDNF*, and *TMEM18.* The reverse analysis was dominated by instruments in the *APOE/TOMM40* region, along with other known AD-risk genes such as *BIN1*, *CLU*, and *PVRL2.* • T2D and Dementia/AD: Forward analysis revealed SNPs in genes related to glucose metabolism (*GIPR*, *ATP6V1G2*) and immune pathways (*PSORS1C1*). In the reverse direction, the *APOE/TOMM40* locus was again central, with additional implicated genes like *RELB* and *PVRL2*, suggesting complex interactions between neurodegeneration and glucose homeostasis.

Our network-informed MR analysis partitioned the causal landscape into six functionally distinct modules, uncovering a complex architecture of risk-conferring, protective, and bidirectional relationships (Figure 2A; Supplementary Table S3). A comprehensive summary of each module is presented in Table 3, while the main text below highlights the key biological narratives, supported by their most representative statistical evidence.

**TABLE 3:** Summary of causal modules linking metabolic and neurocognitive phenotypes identified by network-informed mendelian randomization.

Module ID	Core biological theme	Key causal relationship	Key biological Takeaway	Representative effect (OR [95% CI])	P-value
1	Contrasting effects of hypertension on Dementia	Early-onset hypertension → ↓ Lewy body Dementia; early-onset hypertension → ↑ Atypical/Mixed AD	Hypertension exhibits subtype-specific effects, conferring strong protection against one form of dementia while dramatically increasing risk for another, suggesting distinct underlying vascular pathologies.	1.71 × 10^−8^ [1.86 × 10^−14^–0.016]; 5.66 × 10^8^ [3.05–1.05 × 10^17^]	0.011; 0.038
2	Protective effects of metabolic traits on familial AD	Obesity → ↓ parental history of AD; T2D → ↓ parental history of AD	Genetic predisposition to obesity and T2D is paradoxically associated with a reduced risk of having a parental history of AD, hinting at complex genetic interactions or survival biases.	0.94 [0.89–0.996]; 0.96 [0.93–0.994]	0.035; 0.020
3	Nuanced and bidirectional Obesity-AD links	Class 2 obesity → ↑ AD risk; childhood obesity → ↓ AD risk	The effect of obesity on AD is context-dependent: Severe adult obesity slightly increases risk, while childhood obesity is protective, suggesting different mechanisms related to timing and severity.	1.0005 [1.0002–1.0009]; 0.52 [0.29–0.93]	0.0035; 0.028
4	Consistent protective effect of obesity on AD	Obesity/Hyperalimentation → ↓ AD risk	Multiple genetic definitions of obesity consistently show a protective causal effect against developing AD, challenging the conventional view of obesity as a universal risk factor.	0.90 [0.86–0.95]	1.12 × 10^−5^
5	Potent hypertension risk for early-onset AD	Essential hypertension → ↑ AD risk	A strong, specific causal link exists where primary hypertension dramatically increases the risk of early-onset AD, highlighting a critical role for vascular health in this aggressive form of the disease.	32.17 [1.42–729.21]	0.029
6	Bidirectional T2D-Dementia interplay	T2D → ↑ all-cause Dementia; AD → ↓ T2D risk (protective)	Reveals a complex feedback loop: while T2D is a causal risk factor for dementia, the genetic liability for AD, in turn, confers a protective effect against developing T2D.	1.14 [1.07–1.21]; 0.96 [0.94–0.98]	3.29 × 10^−5^; 3.36 × 10^−5^

The causal influence of hypertension proved to be remarkably potent and context-dependent. It emerged as a potential causal risk factor for early-onset alzheimer’s disease (AD), increasing the risk over 30-fold (Module 5; ukb-b-12493 → finn-b-AD_EO_EXMORE; OR = 32.17; 95% CI: 1.42–729.21; *P* = 0.029). While the primary IVW analysis yielded a notably high odds ratio (OR = 32.17), the extremely wide confidence intervals (95% CI: 1.42–729.21) indicate substantial statistical uncertainty. This magnitude likely reflects the “winner’s curse” phenomenon often observed in MR analyses when exposure and outcome sample sizes are vastly disproportionate (Burgess et al., 2019). Therefore, we interpret this not as a precise clinical risk estimate, but as a robust qualitative signal indicating that early vascular dysregulation significantly elevates AD trajectory. Yet, it also exhibited a paradoxical, subtype-specific effect, simultaneously conferring profound protection against Lewy Body Dementia (Module 1; ieu-b-5142 → ebi-a-GCST90001390; OR = 1.71 × 10^−8^; *P* = 0.011) while dramatically increasing the risk for atypical or mixed AD (ieu-b-5142 → finn-b-AD_AM_EXMORE; OR = 5.66 × 10^8^; *P* = 0.038). These findings strongly suggest that the impact of vascular health on neurodegeneration is highly specific to the underlying dementia pathology.

Our findings on obesity challenged its conventional role as a straightforward risk factor. Across multiple genetic definitions, obesity consistently demonstrated a protective causal effect against AD, with the most significant association observed for obesity with hyperalimentation (Module 4; finn-b-E4_OBESITY_HYPER → ebi-a-GCST90027158; OR = 0.90; 95% CI: 0.86–0.95; *P* = 1.12 × 10^−5^). This protective signal was further nuanced by a context-dependent relationship where childhood obesity was strongly protective (Module 3; ieu-a-1096 → finn-b-AD_U_EXMORE; OR = 0.52; 95% CI: 0.29–0.93; *P* = 0.028), while severe class 2 adult obesity conferred a marginal but statistically significant risk (ieu-a-91 → ieu-b-5067; OR = 1.0005; 95% CI: 1.0002–1.0009; *P* = 0.0035). Together, these results indicate that the timing and nature of adiposity are critical determinants of its effect on neurocognitive health.

Perhaps the most compelling evidence for systemic interplay was found in Module 6, which uncovered a significant bidirectional feedback loop between Type 2 Diabetes (T2D) and dementia. While genetic liability for T2D consistently increased the risk for all-cause dementia (ebi-a-GCST90013892 → finn-b-F5_DEMENTIA; OR = 1.14; 95% CI: 1.07–1.21; *P* = 3.29 × 10^−5^), a reverse causal effect was also robustly evident: a genetic predisposition for AD was, in turn, protective against the development of T2D (finn-b-G6_ALZHEIMER_EXMORE → ebi-a-GCST90013942; OR = 0.96; 95% CI: 0.94–0.98; *P* = 3.36 × 10^−5^). This reciprocal relationship points to a deeply integrated, shared pathophysiology between central glucose metabolism and neurodegenerative processes (Figures 2A,B; Supplementary Table S4).

In addition to these core modules, our analysis identified a protective causal effect of dementia liability on the risk of NAFLD. Genetic liability for a paternal history of AD/Dementia (*ukb-b-323*), for instance, was associated with a striking reduction in the odds of imaging-defined NAFLD (*ebi-a-GCST90054782*) (OR = 0.0007; 95% CI: 3.22 × 10^-5^–0.014; *P* = 2.26 × 10^−6^). This protective signal was consistent across multiple dementia definitions, including “Any dementia” (*finn-b-KRA_PSY_DEMENTIA*) (OR = 0.84; 95% CI: 0.80–0.89; *P* = 2.49 × 10^−10^). While this dementia-to-NAFLD protective link represents a highly significant finding, its unidirectional nature places it in a distinct mechanistic category from the reciprocally-linked metabolic-neurocognitive axes that form the core of our investigation. To maintain a focused analytical scope on these bidirectional feedback loops, which we hypothesized represent the most tightly integrated shared pathologies, we prioritized the symmetric relationships for our initial multi-omics integration. This approach allows for a coherent dissection of the shared molecular architecture underlying these specific reciprocal effects. However, we acknowledge that the protective effect on NAFLD is a critical observation that highlights a potentially distinct biological pathway. It was therefore set aside not due to a lack of importance, but as a key finding that warrants a dedicated, separate investigation to unravel its unique molecular drivers, which may differ from the shared mechanisms explored here. All MR and sensitivity analysis results are presented in Supplementary Tables S5–S8.

## Identification of key genetic loci driving causal associations

To dissect the molecular underpinnings of these causal relationships, we investigated the genetic instruments and their annotated genes, providing insights into the genetic architecture mediating the interplay between metabolic disorders and neurocognitive disorder.

For Hypertension and AD/Dementia associations, forward-direction analysis (metabolic exposure to neurodegenerative outcome) identified instrumental variants, including SNPs such as *rs1052486*, *rs1077394*, and *rs750332*, mapping to genes *ABHD16A*, *AIF1*, and *APOM*. Additional loci included *rs115740542* and *rs198851* (associated with *HFE* and *HIST1H1E*) and *rs12567136* and *rs149764880* (linked to *MTHFR* and *NPPA*). In the reverse direction (neurodegenerative exposure to metabolic outcome), genetic instruments primarily converged on the *APOE/TOMM40* locus, with SNPs such as *rs429358*, *rs769449*, and *rs2972558*. Other implicated genes included *PVRL2* (*rs2972558*) and *BCAM* (*rs2972558*), suggesting roles for lipid metabolism and neuronal adhesion pathways.

For Obesity and AD/Dementia associations, forward-direction analysis implicated obesity-related loci, including variants within or near the *FTO* gene (*rs11075989*, *rs1121980*, *rs1558902*) and the *BDNF* gene (*rs11030104*, *rs11030112*, *rs2030323*). Other genes included *TMEM18* (*rs12623218*, *rs13393304*), frequently associated with body mass index. In the reverse direction, instruments were predominantly in the *APOE/TOMM40* region (*rs112019714*, *rs117310449*, *rs190651665*), with additional genes *BIN1* (*rs6733839*), *CLU* (*rs1532277*, *rs867230*), and *PVRL2* (multiple SNPs), indicating genetic interplay between *AD* and obesity.

For T2D and AD/Dementia associations, forward-direction analysis revealed SNPs in genes related to glucose metabolism, such as *GIPR* (*rs10408179*, *rs10420309*) and *ATP6V1G2* (*rs2857605*, *rs3130924*). Additional instruments were near *PSORS1C1* (*rs1065461*, *rs2233978*, *rs3132520*) and *VNN2* (*rs7773338*). In the reverse direction, the *APOE/TOMM40* locus was central (*rs429358*, *rs112784534*, *rs769449*), with additional genes *RELB* (*rs35080293*, *rs6509191*) and *PVRL2* (multiple SNPs), suggesting complex interactions between neurodegenerative pathways and glucose homeostasis (Figure 2C; Supplementary Table S9).

### Genetic drivers and their recurrence in causal networks

To elucidate the genetic underpinnings of the causal networks between metabolic disorders and neurocognitive disorder, we conducted an intersection analysis of instrumental SNPs and prioritized them by recurrence frequency across significant MR tests. This analysis revealed both unique and shared genetic drivers, highlighting asymmetry in the robustness of genetic signals across forward and reverse causal directions.

In the forward direction (metabolic disorders to AD/Dementia), large, distinct SNP sets were identified: 496 unique SNPs supported the hypertension-to-AD/Dementia network (*e.g.*, *rs74661587*, *rs12258967*), 478 supported the T2D-to-AD/Dementia network (*e.g.*, *rs10830963*, *rs2796441*), and 39 supported the obesity-to-AD/Dementia network (*e.g.*, *rs7138803*, *rs4854344*). The T2D-to-AD/Dementia network exhibited the most robust genetic signal, with highly recurrent SNPs including *rs10830963* (frequency = 79), *rs2796441* (frequency = 74), and *rs11708067* (frequency = 69), averaging 63.5 for the top 10 SNPs. In contrast, the obesity-to-*AD/Dementia* network had a top SNP, *rs7138803* (frequency = 8), and the hypertension-to-*AD/Dementia* network had *rs74661587* (frequency = 5), indicating less concentrated genetic signals.

**FIGURE 3:**
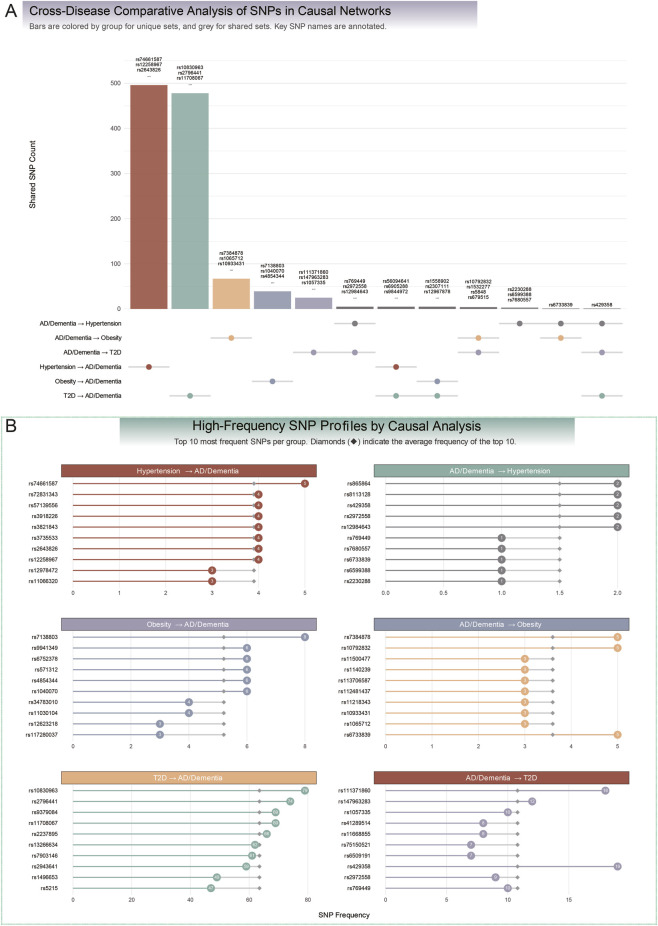
Genetic architecture of causal networks: shared snps and high-frequency drivers. **(A)** A cross-disease comparative analysis of instrumental SNPs from the six primary causal pathways. The bar chart quantifies the number of SNPs unique to each causal pathway (colored bars) and those shared between pathways (grey bars). In the forward direction, large sets of unique SNPs support the causal links from Hypertension to AD/Dementia (496 SNPs) and T2D to AD/Dementia (478 SNPs). In contrast, the reverse direction pathways are supported by smaller SNP sets. The grey bars highlight key pleiotropic SNPs, such as rs429358, which is common to the reverse causal effects of AD/Dementia on both hypertension and T2D. **(B)** High-frequency SNP profiles for each causal direction, displaying the top 10 most recurrent SNPs. The frequency of each SNP is noted within the circle, and the diamond marker indicates the average frequency of the top 10. The analysis reveals a highly concentrated genetic signal for the T2D-to-AD/Dementia network, driven by exceptionally frequent SNPs like rs10830963 (frequency = 79) and rs2796441 (frequency = 74). In contrast, the forward signals from hypertension and obesity, as well as all reverse-direction signals, are more diffuse, characterized by SNPs with much lower recurrence frequencies. These findings highlight a strong, concentrated genetic basis for T2D-driven neurodegeneration and a more heterogeneous genetic architecture for other causal relationships.

In the reverse direction (AD/Dementia to metabolic disorders), smaller SNP sets were observed: 67 for obesity (*e.g.*, *rs7384878*, *rs10933431*), 25 for *T2D* (*e.g.*, *rs111371860*, *rs147963283*), and 3 for hypertension (*e.g.*, *rs2230288*, *rs6599388*, *rs7680557*). The AD/Dementia-to-*T2D* network showed the highest recurrence, with *rs429358* (*APOE*, frequency = 19) and *rs111371860* (frequency = 18). The AD/Dementia-to-obesity network included SNPs like *rs10792832*, *rs6733839* (*BIN1*), and *rs7384878* (frequency = 5). The AD/Dementia-to-hypertension network was the most diffuse, with *rs12984643* and *rs429358* (*APOE*) each at a frequency of 2 (Figure 3A).

Pleiotropic loci underscored shared mechanisms across networks. *rs429358* was common to AD/Dementia effects on hypertension and T2D, and the reciprocal T2D-to-AD/Dementia network. *rs6733839* linked AD/Dementia to hypertension and obesity. Five SNPs, including *rs769449* and *rs2972558*, were shared in reverse effects on hypertension and T2D, while five others, including *rs56094641* and *rs6905288*, connected forward effects of hypertensio*n and T2D* on dementia. Four SNPs, including *rs10792832* and *rs1532277*, linked AD/Dementia to obesity and T2D, and five, including *rs1558902* and *rs12967878*, connected obesity and T2D to AD/Dementia. These findings highlight a concentrated genetic signal for T2D-driven neurodegeneration and a more heterogeneous basis for reverse effects (Figure 3B; Supplementary Table S10).

### Comparative performance of diagnostic models across multiple disease cohorts

To develop biologically informed diagnostic models, we identified gene sets representing key biological pathways implicated in the causal networks linking metabolic and neurocognitive disorder. This process involved multiple steps: first, we prioritized genetic instruments that were both part of robust, network-supported causal links and recurrent across multiple such links. Next, we mapped these SNPs to their putative effector genes using positional mapping. Finally, gene set enrichment analysis against the MSigDB C5 ontology database identified 121, 174, and 158 significantly enriched biological gene sets for the Hypertension-Dementia, Obesity-Dementia, and T2D-Dementia causal networks, respectively (Supplementary Table S11). To evaluate the diagnostic potential of these gene sets, we trained and tested ten machine learning models on six disease-specific transcriptomic datasets, assessing performance *via* the AUC. Model performance varied significantly across diseases and algorithmic architectures, revealing a clear hierarchy in the predictive power of gene expression signatures.

In neurodegenerative cohorts, particularly VaD and AD, models achieved exceptional discrimination. For VaD, the FT-Transformer led with an AUC of 0.908, followed closely by the Support Vector Machine (SVM) (AUC = 0.900) and ExtraTrees (AUC = 0.899). Similarly, in the AD cohort, the FT-Transformer excelled (AUC = 0.874), with SVM (AUC = 0.825) and ExtraTrees (AUC = 0.809) also demonstrating strong predictive utility. For Obesity and T2D, models showed moderate predictive power, with the FT-Transformer again outperforming others, achieving AUCs of 0.804 for *Obesity* and 0.662 for T2D. In contrast, gene signatures for general Dementia and Hypertension exhibited limited diagnostic potential, with the best models—FT-Transformer for Dementia (AUC = 0.594) and Logistic Regression for Hypertension (AUC = 0.598)—performing only marginally better than random chance.

**FIGURE 4:**
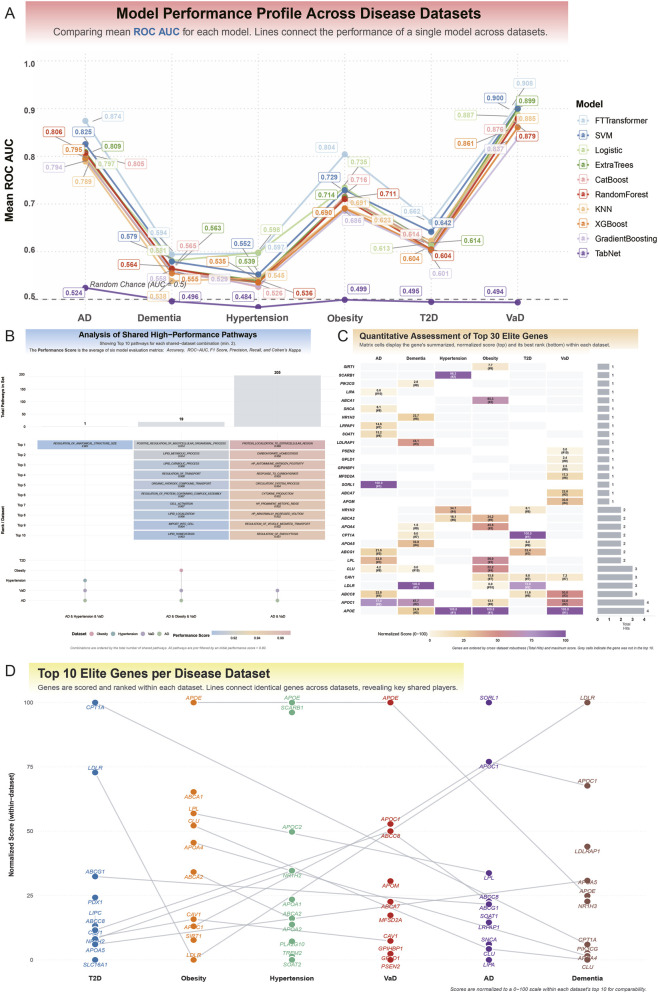
Predictive modeling and multi-omics integration identify key genes and pathways in metabolic and neurocognitive disorder. **(A)** Comparative performance profile of nine machine learning models across six disease datasets, evaluated by mean Receiver Operating Characteristic Area Under the Curve (ROC AUC). The analysis demonstrates exceptional predictive accuracy for neurodegenerative cohorts, particularly Vascular Dementia (VaD) and Alzheimer’s Disease (AD). For VaD, the FT-Transformer model achieved a leading AUC of 0.908, closely followed by Support Vector Machine (SVM) (AUC = 0.900) and ExtraTrees (AUC = 0.899). Similarly, in the AD cohort, the FT-Transformer excelled with an AUC of 0.874. Models for metabolic disorders showed moderate to strong performance, with the FT-Transformer achieving an AUC of 0.804 for Obesity but a more modest 0.662 for T2D. In contrast, gene signatures for general Dementia (top AUC = 0.594) and Hypertension (top AUC = 0.598) showed limited diagnostic potential, performing only marginally above the random chance baseline (AUC = 0.5). The FT-Transformer consistently emerged as the superior model, while TabNet consistently underperformed across all datasets. **(B)** Upset plot analysis identifying shared high-performance biological pathways across disease combinations. This analysis reveals significant overlap in the predictive gene sets, pointing to common molecular mechanisms. The strongest overlap was identified between AD and VaD, with 205 shared pathways, led by “PROTEIN LOCALIZATION TO EXTRACELLULAR REGION” (performance score = 0.969) and “CARBOHYDRATE HOMEOSTASIS” (score = 0.958). A set of 19 pathways was shared among AD, Obesity, and VaD, with top performers including “POSITIVE REGULATION OF MULTICELLULAR ORGANISMAL PROCESS” and “LIPID METABOLIC PROCESS” (both score = 0.914). These findings underscore that shared vulnerabilities in protein localization, carbohydrate homeostasis, and lipid metabolism link these distinct age-related disorders. **(C)** Quantitative assessment and ranking of the top 30 elite genes based on a Holistic Gene Score. This composite score integrates performance-weighted importance from all models with a robustness factor (“Total Hits”) representing the frequency of a gene’s selection across all analyses. The results reveal two distinct gene archetypes: (1) high-impact “specialist” genes, such as *SORL1* in AD (top-ranked with only 1 hit) and *CPT1A* in T2D (highly ranked with 2 hits), which derive their importance from potent, context-specific roles; and (2) broadly influential “generalist” genes, like *APOE* and *APOC1* (each with 4 hits across AD and VaD), which gain prominence through consistent involvement across multiple disease contexts and predictive models. **(D)** Prioritization of the top 10 elite genes within each disease dataset using a Dataset-Specific Score. This score aggregates a gene’s weighted importance and appearance frequency within a single disease context. Lines connect identical genes across datasets, revealing key pleiotropic players. The analysis shows a striking convergence on lipid metabolism. *APOE* stands out as a highly pleiotropic gene, ranking first for VaD, Obesity, and Hypertension. *APOC1* and *CLU* are core components of a shared neurodegenerative signature across AD, Dementia, and VaD. Disease-specific leaders, such as *SORL1* in AD and *CPT1A* (fatty acid oxidation) in T2D, are also identified. This reinforces the central role of dysregulated lipid metabolism as a shared biological mechanism linking these metabolic and Neurocognitive Disorder.

Cross-model comparisons highlighted the consistent superiority of the deep learning FT-Transformer, which was the top performer in five of six datasets and highly competitive in the sixth. A second tier of effective models included SVM and tree-based ensemble methods (ExtraTrees and CatBoost), which provided robust predictions across most datasets. Simpler models, such as Logistic Regression and KNN, offered reliable baseline performance but rarely excelled. Notably, the TabNet model consistently underperformed, with AUCs approximating random chance (≈0.5) across all datasets (Figure 4A; Supplementary Table S12).

Pairwise statistical comparisons confirmed significantly higher predictive accuracy in neurodegenerative cohorts (AD, VaD) compared to metabolic disorders (*P* < 0.0001). The SVM demonstrated robust and balanced performance, significantly outperforming ensemble models like RandomForest in accuracy for the AD cohort (*P* < 0.0001). The FT-Transformer exhibited a specialized advantage in class discrimination and sensitivity, achieving significantly higher AUC (*P* < 0.0001) and recall (*P* < 0.0001) compared to other models in the AD dataset (Supplementary Figures S1 and S2; Supplementary Table S13). These findings underscore the disease-specific diagnostic potential of transcriptomic signatures and the FT-Transformer’s consistent superiority as a classification framework.

### Identification of predictive gene sets for disease classification

Our pathway-centric machine learning approach identified gene sets with robust predictive utility for classifying disease states, revealing both disease-specific and shared biological pathways. The diagnostic performance of these gene sets varied across diseases, reflecting distinct transcriptomic signatures underlying metabolic and neurodegenerative conditions.

For VaD, numerous gene sets exhibited exceptional discriminatory power, achieving AUC values exceeding 0.98 across all metrics. These high-performing gene sets, derived from causal links involving Hypertension, Obesity, and T2D, were functionally diverse. Key examples include *CARBOHYDRATE HOMEOSTASIS* (from the T2D→Dementia network), *AMYLOID PRECURSOR PROTEIN METABOLIC PROCESS* (Obesity→Dementia), and *VASCULAR PROCESS IN CIRCULATORY SYSTEM* (Hypertension→Dementia). These pathways, linked to glucose regulation, amyloid processing, and vascular function, respectively, underscore the strong transcriptomic signatures driving VaD classification.

For AD, gene sets consistently delivered high classification performance. The top-performing gene set, *PROTEIN LOCALIZATION TO EXTRACELLULAR REGION* (T2D→Dementia), achieved an AUC of 0.943 and an accuracy of 0.944, followed closely by *POSITIVE REGULATION OF MULTICELLULAR ORGANISMAL PROCESS* (Obesity→Dementia), with identical AUC and accuracy of 0.943 and 0.944. Other notable gene sets included *CELL ACTIVATION* (immune function), *REGULATION OF TRANSPORT*, and *PHOSPHOLIPID TRANSPORT* (cellular transport and metabolism), all with AUCs exceeding 0.91, highlighting the role of extracellular protein dynamics and immune regulation in AD diagnostics.

In metabolic cohorts, gene sets showed moderate predictive performance. For Obesity, the most predictive signatures, primarily from the reverse Dementia→Obesity network, were linked to lipid biology. The top gene set, *LIPID METABOLIC PROCESS*, achieved an AUC of 0.890 and an accuracy of 0.890, followed by *LIPID LOCALIZATION* (AUC = 0.916) and *REGULATION OF KETONE METABOLIC PROCESS* (AUC = 0.860). For Hypertension, *REGULATION OF ANATOMICAL STRUCTURE SIZE* (Hypertension→Dementia) was the most predictive, with an AUC of 0.863 and an accuracy of 0.856. For T2D, *PROTEIN LOCALIZATION TO EXTRACELLULAR REGION* (T2D→Dementia) yielded an AUC of 0.807 and an accuracy of 0.808. In the general Dementia cohort, where predictive signals were weaker, *REPRODUCTIVE SYSTEM DEVELOPMENT* (Hypertension→Dementia) performed best, with an AUC of 0.698 (Supplementary Figure S3; Supplementary Table S14).

Intersection analysis of high-performing gene sets revealed pleiotropic pathways with predictive utility across multiple diseases. *REGULATION OF ANATOMICAL STRUCTURE SIZ*E was a shared signature across AD, Hypertension, and VaD (overall score = 0.901). A group of 19 gene sets was shared among AD, Obesity, and VaD, with top-ranked pathways including *POSITIVE REGULATION OF MULTICELLULAR ORGANISMAL PROCESS* and *LIPID METABOLIC PROCESS* (both with overall score = 0.914). The strongest overlap occurred between AD and VaD, with 205 shared predictive gene sets, led by *PROTEIN LOCALIZATION TO EXTRACELLULAR REGION* (overall score = 0.969) and *CARBOHYDRATE HOMEOSTASIS* (overall score = 0.958). These results highlight disease-specific transcriptomic signatures alongside shared pathways related to metabolism, protein localization, and organismal regulation, reflecting common biological vulnerabilities across these age-related disorders (Figure 4B).

### Cross-disease prioritization of key genes reveals specialist and generalist signatures

To systematically prioritize the most impactful genes across metabolic and neurocognitive disorder, we developed a Holistic Gene Score. This score aggregates performance-weighted importance scores from all models and pathways, multiplied by a robustness factor derived from the logarithm of a gene’s total appearances (Total_Hits), which reflects the number of times a gene was identified as a predictive feature across all models, pathways, and disease datasets. This unified framework balances raw predictive power with consistency across diverse biological and analytical contexts. Analysis of contribution patterns revealed two gene archetypes: high-impact “specialist” genes and broadly influential “generalist” genes. Specialist genes, such as *SORL1* in AD (Total_Hits = 1), achieved top rankings through exceptional influence in specific predictive pathways. Similarly, *LDLR* in general Dementia (Total_Hits = 3) and *CPT1A* in T2D (Total_Hits = 2) secured high ranks *via* limited but potent contributions. In contrast, generalist genes like *APOE* and *APOC1*, each identified in four contexts (Total_Hits = 4) for AD and VaD, and *CAV1* across three contexts (AD, Obesity, VaD), gained prominence through consistent involvement across multiple models and pathways. This distinction highlights that a gene’s significance in a disease’s transcriptomic signature can arise from either context-specific potency or widespread influence across biological processes (Figure 4C).

For disease-specific prioritization, we calculated a Dataset-Specific Score for each gene by aggregating performance-weighted importance scores from all models and pathways, weighted by the logarithm of appearance frequency. This approach prioritized genes with consistent importance across high-performing models and frequent roles in distinct pathways. The top 10 genes per disease were selected based on this score, revealing both disease-specific and shared signatures, with a notable convergence on lipid metabolism and transport pathways. In AD, *SORL1* topped the rankings, followed by lipid metabolism genes *APOC1*, *LPL*, *ABCG1*, and *LIPA*. For VaD, *APOE* and *APOC1* led, underscoring lipid transport’s critical role. General Dementia signatures were dominated by *LDLR* and *APOC1*, followed by *LDLRAP1* and *APOA5*. The consistent prominence of *APOC1* and *CLU* across dementia-related cohorts highlights their role as core components of a shared neurodegenerative signature. In metabolic cohorts, Obesity was led by *APOE*, followed by cholesterol transport and lipid metabolism genes *ABCA1*, *LPL*, and *CLU*, overlapping significantly with dementia signatures. For Hypertension, *APOE* and *SCARB1* topped the list, alongside inflammatory response gene *TREM2*. The T2D signature was distinct, with *CPT1A* (fatty acid oxidation) ranked highest, followed by cholesterol homeostasis genes (*LDLR*, *ABCG1*) and insulin secretion gene *ABCC8*. Cross-disease comparison identified *APOE* as a highly pleiotropic gene, ranking first for VaD, Obesity, and Hypertension, and in the top five for Dementia. Similarly, *APOC1*, *CLU*, and *LPL* consistently ranked among top-tier genes across both neurodegenerative and metabolic disorders, reinforcing lipid metabolism dysregulation as a shared biological mechanism linking these age-related diseases (Figure 4D). All gene importance rankings are presented in Supplementary Table S15.

### Lipid dysregulation as a central molecular axis and cross-disease pathway activation

Analysis of top-ranked genes and their pathway contributions revealed a molecular landscape consistently dominated by lipid dysregulation across all six diseases. Pathways related to lipid and lipoprotein metabolism were among the strongest contributors, exemplified by *APOE* and *APOC1* in the VaD cohort (Summarized Scores = 1,225.30 and 949.91, respectively) and *LDLR* in the dementia cohort (Score = 502.57). Cross-disease pathway activation emerged in the obesity cohort, where the transcriptomic signature intersected with hallmark processes of neurodegeneration. While the top gene *ABCA1* (Score = 250.50) was driven by the expected *IMPORT INTO CELL* pathway, the second-ranked gene *APOE* (Score = 246.74) derived its major contribution from the *TAU PROTEIN BINDING* pathway, a molecular feature central to AD. Conversely, the AD signature itself blended classic neurodegenerative and metabolic processes: *SORL1* (Score = 648.53) was uniquely linked to *NEUROFIBRILLARY TANGLES*, whereas *APOC1* (Score = 537.59) and *SOAT1* (Score = 398.41) were primarily associated with *NEGATIVE REGULATION OF LIPID TRANSPORT* and *REGULATION OF PLASMA LIPOPROTEIN PARTICLE ASSEMBLY*.

**FIGURE 5:**
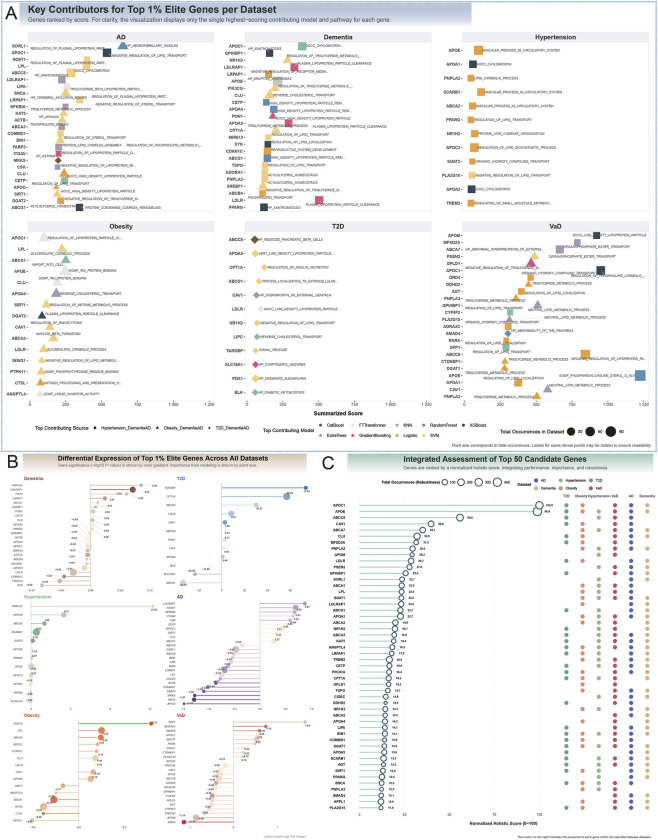
Functional annotation and multi-omics characterization of elite genes linking metabolic and neurocognitive disorder. **(A)** Top-ranked elite genes and their primary contributing pathways for each of the six disease datasets. For clarity, only the single highest-scoring model and pathway contribution is displayed for each gene. The analysis reveals a molecular landscape consistently dominated by lipid dysregulation, with pathways related to lipoprotein metabolism, such as *REGULATION OF PLASMA LIPOPROTEIN PARTICLE ASSEMBLY* and *NEGATIVE REGULATION OF LIPID TRANSPORT*, consistently contributing top scores. This is exemplified in the VaD cohort by *APOE* (Summarized Score = 1,225.3) and *APOC1* (Score = 949.9). Significant molecular crosstalk is evident, as seen in the Obesity cohort, where the score for the second-ranked gene, *APOE* (Score = 246.7), is driven by the *TAU PROTEIN BINDING* pathway, a molecular hallmark of AD. Conversely, the AD signature represents a blend of neurodegenerative pathways (e.g., *SORL1*, Score = 648.5, linked to *NEUROFIBRILLARY TANGLES*) and metabolic pathways (e.g., *APOC1*, Score = 537.6, linked to *NEGATIVE REGULATION OF LIPID TRANSPORT*), underscoring complex inter-disease molecular mechanisms. **(B)** Differential expression analysis (log-fold change) of the top elite genes across all six datasets. This analysis provides transcriptional evidence supporting the pathogenic mechanisms identified by predictive modeling. In neurodegenerative cohorts, genes involved in lipid and amyloid clearance are broadly downregulated, including *SORL1* in AD (logFC = −0.58, P = 0.002) and *LDLR* in Dementia (logFC = −0.62, P = 0.0005). In metabolic disorders, crosstalk-related genes show distinct regulatory patterns: *APOE* is significantly upregulated in Obesity (logFC = +0.55, P = 0.009), while *CPT1A* is significantly downregulated in T2D (logFC = −0.71, P = 0.0001), consistent with mitochondrial dysfunction. Furthermore, the analysis identifies shared dysregulation of genes involved in systemic processes: *ABCC8* (energy sensing) is downregulated in AD (logFC = −0.42) and VaD (logFC = −0.51); *NFKBIA* (inflammation) is downregulated in AD (logFC = −0.37); and *ABCG1* (cholesterol efflux) is downregulated in both AD (logFC = −0.49) and T2D (logFC = −0.31), pointing to systemic impairment of these critical biological axes. **(C)** Integrated assessment and holistic ranking of the top 50 candidate genes. Genes are ranked by a normalized holistic score that integrates predictive performance, pathway importance, and robustness (total occurrences across all analyses). *APOC1* (Holistic Score = 11202.4) and *APOE* (Score = 11075.5) emerge as the top-ranked pleiotropic candidates, implicated across five and six disease cohorts, respectively. Beyond canonical neurodegeneration-associated genes like *CLU* (Rank = 6), the ranking highlights novel, highly connected molecular nodes that bridge metabolic and neurodegenerative pathologies. These include *CAV1* (Rank = 4, Score = 4453.7), involved in insulin signaling and lipid raft dynamics; *MFSD2A* (Rank = 7, Score = 3528.4), responsible for omega-3 transport across the blood–brain barrier; and *PNPLA2* (Rank = 8, Score = 3305.9), the rate-limiting enzyme for triglyceride hydrolysis. These findings identify novel, high-priority targets for future mechanistic and therapeutic research.

A similar trend was observed in T2D, where the top gene *CPT1A* (Score = 120.19) was linked to *REGULATION OF INSULIN SECRETION*, yet other contributors, such as *LDLR* (Score = 96.22), implicated lipid clearance mechanisms also prominent in dementia. These findings suggest that disease-specific pathways can emerge as key transcriptomic drivers in unrelated conditions, underscoring complex molecular crosstalk between metabolic and neurodegenerative processes (Figure 5A).

### Overall characteristics of differential gene expression

Differential gene expression analysis unveiled distinct transcriptional dysregulation patterns across the six disease cohorts, varying markedly in both the extent of affected genes and the magnitude of their expression alterations. Neurodegenerative disorders, notably VaD and AD, exhibited the most widespread transcriptional perturbations, with the highest numbers of differentially expressed genes (5,918 downregulated and 5,477 upregulated in VaD; 4,850 downregulated and 3,305 upregulated in AD). Nonetheless, the average magnitude of these changes remained modest, with mean absolute logFC ranging from approximately 0.41–0.61. A comparable yet less extensive profile was evident in Obesity, featuring a substantial number of dysregulated genes (2,998 downregulated and 2,529 upregulated), but with notably smaller average expression shifts (avg. abs. logFC ≈0.16). The general Dementia cohort mirrored this trend of subtle dysregulation, albeit with fewer affected genes (1,603 downregulated and 1,377 upregulated) and the lowest average change magnitude (avg. abs. logFC ≈ 0.03). In contrast, metabolic disorders were typified by more focal yet pronounced gene dysregulation. The T2D cohort demonstrated the most extreme expression alterations, with an exceptionally high average absolute logFC, particularly among the 1,073 downregulated genes (avg. abs. logFC = 52.80). Hypertension presented the most circumscribed transcriptomic signature, involving the fewest differentially expressed genes (91 downregulated and 486 upregulated), but these exhibited substantial magnitudes, especially for downregulated genes (avg. abs. logFC = 3.11) (Supplementary Figure S4).

All DEGs are comprehensively listed in Supplementary Table S16.

### Transcriptomic directionality and shared mechanistic axes

Integration of DEG data revealed consistent transcriptional patterns supporting shared pathogenic mechanisms. In neurodegenerative cohorts, genes involved in lipid and amyloid clearance were broadly downregulated. In AD, *SORL1* was significantly downregulated (logFC = −0.58, *P* = 0.002), accompanied by decreased expression of *APOC1* (logFC = −0.45, *P* = 0.011) and *LPL* (logFC = −0.39, *P* = 0.023). In dementia, *LDLR* was also downregulated (logFC = −0.62, *P* = 0.0005), reinforcing the concept of impaired clearance as a unifying mechanism. In metabolic disorders, crosstalk-related genes exhibited distinct regulatory directions. In obesity, *APOE* was significantly upregulated (logFC = +0.55, *P* = 0.009), supporting its potential involvement in tau-associated pathways. In T2D, *CPT1A* was significantly downregulated (logFC = −0.71, *P* = 0.0001), consistent with mitochondrial fatty acid oxidation deficits and β-cell dysfunction. Similarly, in hypertension, the top-ranked vascular gene *SCARB1* showed downregulation (logFC = −0.33, *P* = 0.041), reflecting impaired vascular cholesterol handling.

Further analysis revealed shared molecular axes beyond lipid transport, encompassing energy sensing, inflammation, and cholesterol efflux. *ABCC8*, a KATP channel subunit essential for energy coupling, ranked highly in AD (Rank = 7), VaD (Rank = 4), and T2D (Rank = 12) and was downregulated in both AD (logFC = −0.42, *P* = 0.015) and VaD (logFC = −0.51, *P* = 0.008), suggesting impaired neuronal and pancreatic energy homeostasis. *NFKBIA*, a key inhibitor of NF-κB signaling, was also top-ranked in AD (Rank = 10) and downregulated (logFC = −0.37, *P* = 0.028), indicative of chronic NF-κB pathway activation—a hallmark of both neuroinflammation and insulin resistance. Similarly, *ABCG1*, a central cholesterol transporter ranked among the top genes in AD (Rank = 6) and T2D (Rank = 3), was consistently downregulated (AD: logFC = −0.49, *P* = 0.009; T2D: logFC = −0.31, *P* = 0.045), pointing to systemic impairment of cholesterol efflux (Figure 5B; Supplementary Table S17).

A holistic ranking combining feature importance and robustness identified a small set of pleiotropic genes as key molecular nodes bridging metabolic dysregulation and neurodegeneration. *APOC1* and *APOE* emerged as the top candidates overall (holistic scores = 11202.41 and 11075.54, respectively), with *APOE* implicated in all six disease cohorts and *APOC1* in five. Canonical neurodegeneration-associated genes such as *CLU* (Rank = 6) were also repeatedly prioritized. Notably, several less-studied but highly connected genes were highlighted, including *CAV1* (Rank = 4, Score = 4453.66), implicated in insulin signaling and lipid raft dynamics; *MFSD2A* (Rank = 7, Score = 3528.35), responsible for omega-3 fatty acid transport across the blood–brain barrier; and *PNPLA2* (Rank = 8, Score = 3305.88), the rate-limiting enzyme for triglyceride hydrolysis. These genes, identified across multiple cohorts, underscore shared mechanisms linking metabolic and neurodegenerative pathology and provide novel targets for future mechanistic and therapeutic research (Figure 5C; Supplementary Table S18).

### Identification of pleiotropic chemicals and their convergence on a core gene network

**FIGURE 6:**
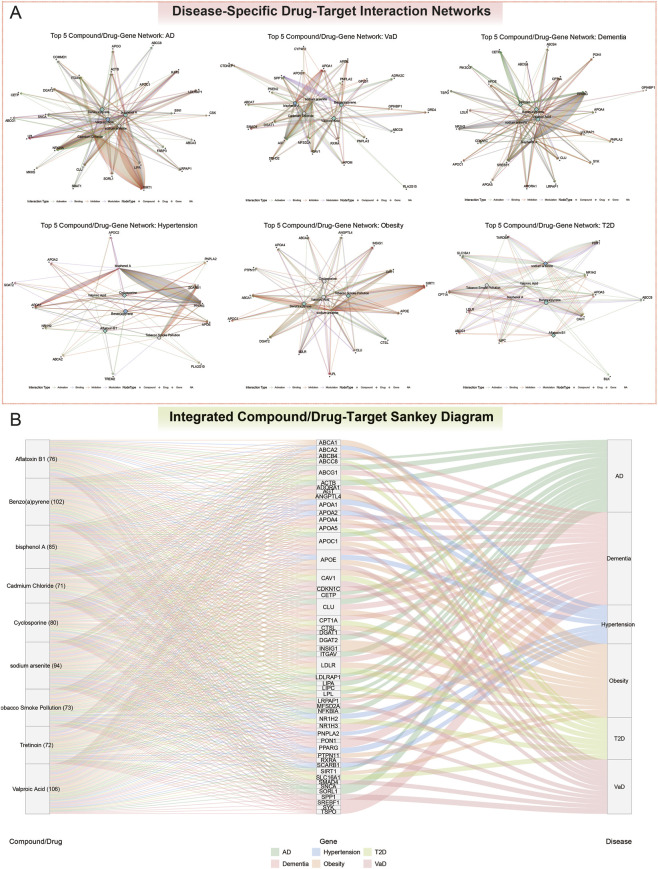
Chemical-gene interaction networks reveal pleiotropic compounds and convergent targeting of core disease genes. **(A)** Disease-specific interaction networks for the top five hub compounds/drugs and their identified target genes within each of the six disease cohorts. The analysis highlights the consistent emergence of highly pleiotropic chemicals across the disease spectrum. Specifically, the therapeutic drug Valproic Acid is identified as the top hub compound for Alzheimer’s Disease (AD), Dementia, and Vascular Dementia (VaD), interacting with 25, 23, and 22 prioritized genes, respectively. The environmental toxicant Benzo(a)pyrene demonstrates similar pleiotropy, serving as the leading hub for Hypertension and T2D (12 targets each) and ranking among the top three for all neurodegenerative cohorts, underscoring its broad biological impact. **(B)** An integrated Sankey diagram visualizing the convergent targeting of a core network of pleiotropic genes by eight high-priority chemicals. These chemicals include six environmental toxicants (Aflatoxin B1, Benzo(a)pyrene, Cadmium Chloride, bisphenol A, sodium arsenite, Tobacco Smoke Pollution) and two therapeutic drugs (Cyclosporine, Valproic Acid). The diagram reveals that a small set of key genes serve as central nodes, receiving interactions from a majority of these compounds. Most prominently, *APOC1*, *APOE*, and *CAV1* are targeted by seven, six, and seven of the eight prioritized chemicals, respectively. Other critical shared targets, including *LDLR* (the top-ranked gene in Dementia), *PNPLA2* (a key VaD-associated gene), and the metabolic regulator *SIRT1*, are each modulated by six of the selected chemicals. This striking convergence suggests that the disruption of shared pathways—particularly those governing lipid metabolism, cellular stress, and metabolic sensing—represents a common molecular mechanism linking the biological effects of these diverse compounds to both metabolic and neurodegenerative disease pathology.

To explore potential therapeutic agents and environmental exposures affecting the prioritized gene signatures, we conducted a chemical enrichment analysis using the CTD database. This approach identified several hub chemicals—compounds interacting with numerous top-ranked genes in each disease cohort. The therapeutic drug Valproic Acid and the environmental toxicant Benzo(a)pyrene consistently emerged as the most pleiotropic chemicals across the disease spectrum. Valproic Acid was the top hub compound for AD (targeting 25 key genes), Dementia (23 targets), and VaD (22 targets). Benzo(a)pyrene showed similar pleiotropy, serving as the top hub for Hypertension (12 targets) and T2D (12 targets), while also ranking among the top three for AD, Dementia, and VaD (Figure 6A).

To prioritize candidates for downstream analysis, we selected eight high-priority chemicals, including six environmental toxicants—Aflatoxin B1 [D016604], Benzo(a)pyrene [D001564], Cadmium Chloride [D019256], bisphenol A [C006780], sodium arsenite [C017947], and Tobacco Smoke Pollution [D014028]—and two therapeutic drugs, Cyclosporine [D016572] and Valproic Acid [D014635]. These chemicals converged on a shared network of highly pleiotropic gene targets, most prominently *APOC1*, *APOE*, and *CAV1*. Seven of the eight chemicals (Aflatoxin B1, Benzo(a)pyrene, Cadmium Chloride, Cyclosporine, Valproic Acid, bisphenol A, and sodium arsenite) targeted *APOC1*, a gene highly ranked in AD (Score = 537.6) and VaD (Score = 949.9). Similarly, six chemicals (Aflatoxin B1, Benzo(a)pyrene, Cyclosporine, Valproic Acid, bisphenol A, and sodium arsenite) targeted *APOE*, the top-ranked gene in VaD (Score = 1,225.3) and an influential gene in Obesity (Score = 246.7). *CAV1*, a key signaling molecule with strong importance in VaD (Score = 579.8) and Obesity (Score = 138.6), was targeted by seven chemicals, including Aflatoxin B1, Benzo(a)pyrene, and Valproic Acid (Figure 6B).

In addition to these core nodes, several other shared targets were modulated by the majority of the prioritized chemicals. *LDLR*, the top gene in Dementia (Score = 502.6), was targeted by Aflatoxin B1, Benzo(a)pyrene, Cadmium Chloride, Cyclosporine, Valproic Acid, and Tobacco Smoke. *PNPLA2*, a VaD-associated gene (Score = 504.3), was modulated by Benzo(a)pyrene, Cadmium Chloride, Valproic Acid, bisphenol A, sodium arsenite, and Tobacco Smoke. Another critical target, *SIRT1*, a metabolic regulator with high importance scores in AD (Score = 192.3) and Obesity (Score = 115.0), was targeted by six chemicals (Cadmium Chloride, Cyclosporine, Valproic Acid, bisphenol A, sodium arsenite, and Tobacco Smoke). These convergent interactions suggest that disruptions of lipid metabolism, cellular stress responses, and metabolic sensing pathways represent common molecular mechanisms connecting neurodegenerative and metabolic disorders (Supplementary Tables S19 and S20).

### Phenotypic and transcriptomic validation of an AD-like murine model

To establish a translationally relevant *in vivo* model, we first confirmed that the D-galactose and sodium nitrite regimen induced a robust neurodegenerative phenotype. Behaviorally, the model cohort (n = 5) exhibited profound cognitive deficits in the Morris Water Maze, including both impaired spatial learning (*P* < 0.001) and deficient memory consolidation (*P* < 0.05), when compared to controls (n = 5). This functional decline was mirrored by a molecular signature of neuroinflammation and synaptic loss within the hippocampus, as evidenced by a significant reduction in the Gri2a/Tnfrsfr11b transcript ratio (*P* < 0.01).

**FIGURE 7:**
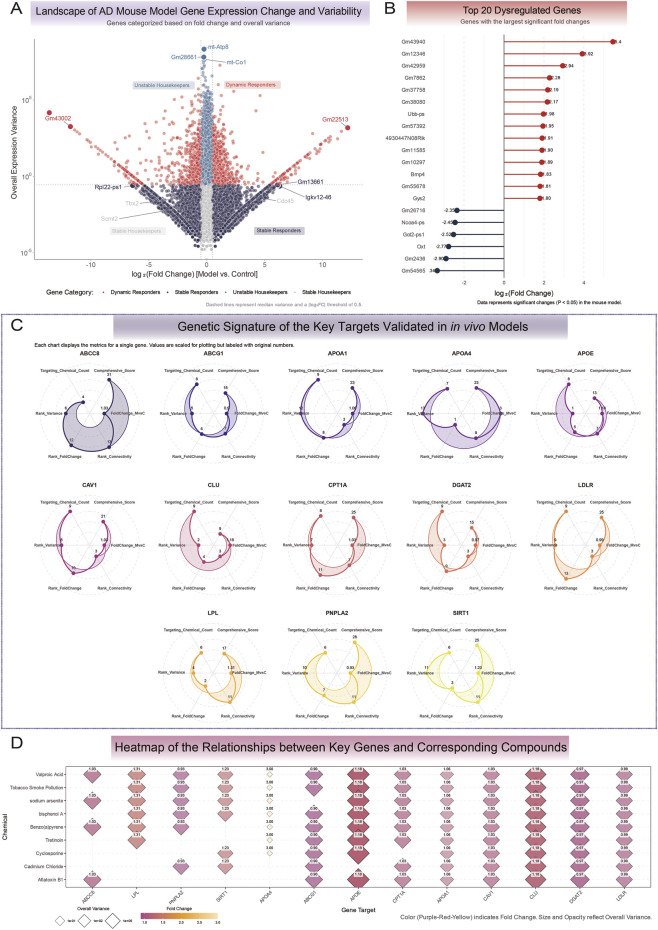
Integrated transcriptomic analysis, target prioritization, and chemical interactome mapping in the ad mouse model. **(A)** A variance-volcano plot illustrates the global landscape of gene expression changes. Out of 1,167 analyzed genes, 110 were identified as significantly differentially expressed (P < 0.05, |log_2_FC| >1.0). Key genes such as Gm43940 (most upregulated) and Igkv6-23 (most statistically significant) are highlighted among the “Dynamic Responders” (red), which exhibit both high fold change and high variance. **(B)** Lollipop chart depicting the top 20 most significantly dysregulated genes. The list includes the most upregulated gene, Gm43940 (log_2_FC = 5.45), and the most downregulated gene, Gm54565 (log_2_FC = −3.34), providing a focused view on the genes with the most profound ex-pression changes in the model. **(C)** Radar charts displaying the multi-parametric genetic signature of the 13 prioritized key tar-gets, which were selected from the pool of DEGs. The color gradient, transitioning from blue to yellow, corresponds to the ranking by Comprehensive_Score. The unique profiles of top-tier candidates CLU (Comprehensive_Score = 9) and APOE (Comprehensive_Score = 13) are shown, highlighting their balanced, high-ranking performance across metrics like Rank_Connectivity and Rank_Variance. The chart for APOA4 illustrates a more specialized profile, distinguished by the best possible Rank_FoldChange (rank 1) due to its profound upregulation (Fold-Change_MvsC = 3.00). **(D)** A heatmap illustrating the experimentally-derived and literature-supported interactions between the 13 prioritized gene targets and a panel of corresponding chemical compounds. The fill color of the diamond nodes (purple-red-yellow) indicates the FoldChange_MvsC, while their size and opacity are proportional to the Overall_Variance. This panel visually confirms the broad activity of agents like Valproic Acid (targeting all 13 genes) and environmental factors like Benzo(a)pyrene (targeting 12 genes). It also highlights specific high-impact interactions discussed in the text, such as the consistent upregulation of APOE by most tested compounds and the potent induction of APOA4 by chemicals including Benzo(a)pyrene and Cyclosporine.

To elucidate the global transcriptional changes driving this phenotype, we performed a comprehensive differential expression analysis on hippocampal RNA. From a total of 1,167 genes analyzed, we identified 110 significantly differentially expressed genes (DEGs) based on the criteria of an uncorrected P-value < 0.05 and an absolute log_2_ fold change >1.0 (Figure 7A; Supplementary Table S21). The transcriptional response was predominantly characterized by upregulation, with 78 genes induced *versus* 32 genes repressed in the model group. The gene exhibiting the most substantial upregulation was *Gm43940* (log_2_FC = 5.45, *P* = 6.34 × 10^−5^), whereas *Gm54565* showed the most significant downregulation (log_2_FC = −3.34, *P* = 2.52 × 10^−2^). From a statistical standpoint, *Igkv6-23* was the most robustly identified DEG (log_2_FC = 1.79, *P* = 1.17 × 10^−6^). The expression patterns of these and other key DEGs are visualized in a volcano plot and heatmap (Figure 7B).

### Integrated prioritization, chemical interactome, and structural validation of key therapeutic targets

From the pool of DEGs, we employed a multi-parametric ranking strategy to prioritize 13 key targets with high therapeutic potential. This Comprehensive_Score, calculated from chemical connectivity, expression modulation, and biological variance, allowed for a holistic assessment of each candidate’s profile (Figure 7C). The analysis identified *CLU* and *APOE* as top-tier candidates due to their balanced, high-ranking profiles across all metrics. However, it also highlighted genes with more specialized strengths; for instance, *APOA4* achieved the best possible Rank_FoldChange due to its profound upregulation (FoldChange_MvsC = 3.00), resulting in a more moderate Comprehensive_Score of 23 despite lower ranks in other categories.

To explore the pharmacological landscape of these prioritized genes, we mapped their interactions with a diverse chemical library (Figure 7D). This revealed that our top-ranked targets are highly connected hubs. For example, our premier target, *CLU*, was modulated by nine distinct chemicals, including the therapeutic agent Tretinoin and the environmental toxin Aflatoxin B1. Similarly, *APOE* was a common target for eight compounds, consistently showing significant upregulation (FoldChange_MvsC = 1.18) in response to agents like Benzo(a)pyrene and bisphenol A. The highly upregulated *APOA4* was also found to be a convergence point for multiple chemical classes, being potently induced by Benzo(a)pyrene, Cyclosporine, and Tretinoin, which validates its role as a key responsive gene.

**FIGURE 8:**
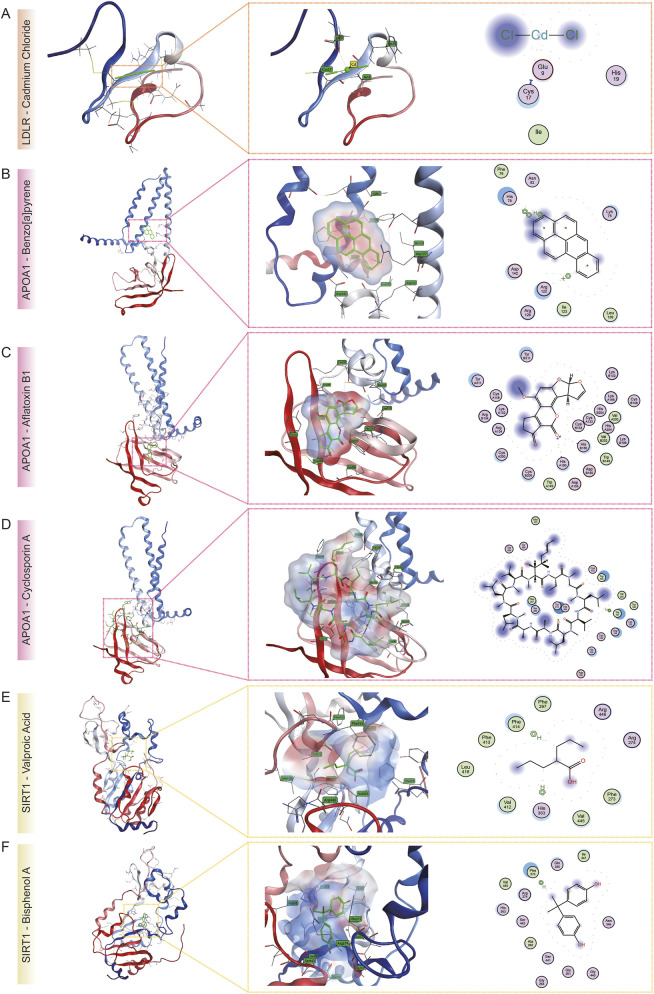
Molecular docking validates high-affinity interactions between prioritized chemicals and key shared protein targets. The figure displays the results of molecular docking simulations for six representative chemical–protein pairs, providing structural evidence for their interactions. Each panel illustrates the predicted binding mode, including a 3D view of the binding pocket with its electrostatic surface potential and a 2D diagram detailing the specific intermolecular interactions (e.g., hydrogen bonds, hydrophobic interactions). • **(A)** The heavy metal Cadmium Chloride shows a weak predicted interaction with *LDLR* (London dG = −3.25 kcal/mol). • **(B–D)** The lipid transport protein *APOA1* is targeted by multiple chemicals. The therapeutic agent Cyclosporine A shows the highest predicted binding affinity (London dG = −8.93 kcal/mol), forming a stable complex. The environmental toxicants Benzo(a)pyrene and Aflatoxin B1 also show favorable binding, with predicted energies of −5.01 kcal/mol and −5.57 kcal/mol, respectively. • **(E, F)** The metabolic regulator *SIRT1* is targeted by both a therapeutic drug and an environmental toxicant. Valproic Acid is predicted to form a stable complex (binding energy = −5.11 kcal/mol), as is the endocrine disruptor bisphenol A (binding energy = −5.32 kcal/mol). Collectively, these structural analyses provide a plausible molecular basis for the identified chemical–gene interactions, supporting the hypothesis that both therapeutic and environmental chemicals can directly engage with a core network of proteins implicated in the pathology of metabolic and Neurocognitive Disorder.

Finally, to provide a structural basis for these interactions, we performed molecular docking simulations (Figure 8; Supplementary Table S22). The results predicted stable, high-affinity binding for our key genes, reinforcing their biological relevance. Supporting the interactome data, Aflatoxin B1 and Benzo(a)pyrene were both predicted to bind a key protein related to *APOA4* and *APOE*’s lipid metabolism pathways, *APOA1*, with significant energies (−5.57 and −5.01 kcal/mol, respectively). Furthermore, Cyclosporine exhibited the highest predicted binding affinity to *APOA1* (London dG = −8.93 kcal/mol), providing a structural rationale for its potent induction of the functionally related *APOA4*. Collectively, this integrated analysis—from data-driven prioritization to chemical interactome and structural modeling—provides a multi-layered line of evidence supporting the central role of targets like *CLU*, *APOE*, and *APOA4* in both therapeutic and pathological neurodegenerative pathways.

## Discussion

This study employs a network-informed MR framework, integrating transcriptomics, chemical perturbation analysis, and molecular docking to explore causal relationships between metabolic disorders and neurocognitive disorder. To ground our computational predictions in a biologically relevant context, we first established an *in vivo* murine model that recapitulates key features of AD-like neurodegeneration. Transcriptomic analysis of this model provided a set of experimentally prioritized gene targets with validated dysregulation in a pathological context, which served as a crucial anchor for our broader systems-level investigation. Rather than treating these as isolated conditions, our analysis reveals interconnected biological mechanisms, with lipid dysregulation emerging as a unifying molecular theme, complemented by subtle but consistent changes in mitochondrial energy regulation.

The findings converge on lipid metabolism as a core driver of disease risk and progression. The recurrent involvement of the *APOE/TOMM40* locus highlights its well-established role in cholesterol transport, lipoprotein assembly, and neuronal membrane maintenance (Huang et al., 2024; Wiegers et al., 2025). Isoform-specific effects, particularly those of *APOE4*, are known to disrupt lipid homeostasis, promote amyloid deposition, and intensify microglial-mediated inflammation (Xu et al., 2020; Haney et al., 2024; Juul Rasmussen et al., 2024). Our results expand this framework by showing that obesity-associated *APOE* upregulation is linked to tau-binding pathways, suggesting that systemic metabolic alterations may prime neuronal vulnerability even before overt neurodegeneration (Qiang et al., 2024). Crucially, the transcriptomic analysis from our *in vivo* model supports reinforces this conclusion, where our multi-parametric ranking identified *APOE* as a top-tier target distinguished by the highest overall variance in the dataset. This experimental finding demonstrates that *APOE* is not only a genetic risk factor but also a highly dynamic and responsive gene within a pathological context.

The consistent downregulation of lipid receptors and trafficking molecules such as *SORL1* in AD and *LDLR* in general dementia further implicates impaired endosomal processing and lipoprotein receptor signaling. These disruptions likely compromise amyloid precursor protein trafficking and cholesterol clearance, processes known to influence amyloid plaque formation (Yin, 2023). Epidemiological evidence linking hyperlipidemia and dysregulated cholesterol transport to cognitive decline aligns with our finding that genetic variants affecting lipid pathways have directional causal effects on dementia risk (Meikle et al., 2020; Zeng et al., 2023).

Partitioning causal associations into six functional modules provides insight into disease-specific heterogeneity. For example, hypertension displayed opposing effects on dementia subtypes, decreasing the risk of Dementia with Lewy bodies while increasing the risk of atypical Alzheimer’s phenotypes (Pasqualetti et al., 2022; Huang et al., 2023), suggesting that vascular and lipid pathway interactions may modify neurodegenerative trajectories differently by subtype. Obesity showed context-dependent protective associations with certain Alzheimer’s endpoints, consistent with emerging hypotheses that adipose-derived cytokines and lipids may exert compensatory effects under specific metabolic states (Gentile et al., 2020; Muddapu et al., 2020). Such heterogeneity supports the need for precision stratification when designing prevention and treatment strategies, recognizing that metabolic risk factors may not uniformly translate into neurodegenerative outcomes (Arjunan et al., 2023; Yang et al., 2023; Wang Z. et al., 2025).

A particularly intriguing finding from our MR analysis was the strong, unidirectional protective effect of dementia liability on the risk of NAFLD. While our downstream multi-omics integration was intentionally focused on dissecting the shared molecular architecture of bidirectional relationships, this unidirectional link warrants specific discussion as it may unveil distinct biological pathways. Several non-mutually exclusive mechanisms could explain this counterintuitive observation. The most direct explanation may relate to the profound metabolic and behavioral changes that often accompany neurodegenerative progression (Yang et al., 2023). Clinical observations consistently document significant weight loss, reduced appetite (anorexia), and altered dietary patterns in individuals with dementia, particularly in later stages. Since weight loss and caloric restriction are the most effective interventions for resolving hepatic steatosis, the catabolic state associated with dementia could directly lead to a reduction in liver fat, thus appearing as a protective effect in genetic analyses (Horie et al., 2016). Alternatively, this association could be driven by antagonistic pleiotropy, where certain genetic variants increase dementia risk while simultaneously promoting a metabolic profile (e.g., enhanced lipid clearance or reduced hepatic lipid uptake) that is protective against NAFLD (Byars and Voskarides, 2020). The *APOE* locus itself, with its complex and opposing effects on lipid metabolism in the central nervous system *versus* the periphery, could be a candidate for such a mechanism (Mahley, 2016). Finally, altered neuroendocrine signaling originating from degenerating brain regions, such as the hypothalamus, could systemically shift metabolic regulation away from fat storage (Procaccini et al., 2016). Future research should aim to disentangle these possibilities through longitudinal studies that correlate cognitive decline with changes in body composition, diet, and liver fat content. Furthermore, Mendelian randomization analyses incorporating genetic instruments for weight change could help test for mediation, while functional studies of pleiotropic loci in both hepatic and neuronal cell models could clarify their tissue-specific effects.

While lipid dysregulation dominated the genetic and transcriptomic signals, several prioritized genes implicate subtle but meaningful changes in cellular energy metabolism. Downregulation of *CPT1A* in T2D, for instance, points to impaired fatty acid β-oxidation and reduced flexibility in energy substrate utilization, which may exacerbate insulin resistance and neuronal energy deficits (Ibeas et al., 2024; Ju et al., 2024). Importantly, the identification of *CPT1A* as a key target with a balanced profile in our *in vivo* screen provides additional support for its relevance in the AD-like state. Similar metabolic bottlenecks have been described in AD, where reduced efficiency of lipid utilization contributes to synaptic vulnerability (Qi et al., 2021; Yuan et al., 2025). The resulting energy deficits are further compounded by mitochondrial dysfunction and impaired mitophagy, which are early features of AD and contribute to synaptic loss and cognitive decline (Cuperfain et al., 2018; Clemente-Suárez et al., 2023). Thus, subtle changes in genes like *CPT1A* highlight the importance of energy metabolism in neurodegenerative disease and suggest new avenues for prevention and treatment targeting metabolic pathways.

In addition, attenuation of *ABCC8*, a key component of ATP-sensitive potassium (KATP) channels, was observed in both AD and VaD, highlighting the importance of these channels as metabolic sensors that couple mitochondrial ATP production to neuronal excitability (Grizzanti et al., 2023; Xiao et al., 2023). Disruption of KATP channel signaling impairs the neuronal response to metabolic stress, making neurons more vulnerable to energy deficits and potentially exacerbating amyloid pathology, as KATP channels regulate the relationship between glucose metabolism, neuronal activity, and amyloid-β release (Abdelkader et al., 2020; Maqoud et al., 2022). Mutations or downregulation of KATP channel subunits can disturb network oscillations, increase seizure risk, and impair cognitive function, further linking metabolic dysfunction to neurodegenerative vulnerability (Burkart et al., 2024). Additionally, the downregulation of *ABCG1*, which mediates cholesterol efflux, may alter mitochondrial membrane composition and oxidative balance, underscoring the intersection of lipid handling and intracellular energy homeostasis (Xu et al., 2024). Both *ABCC8* and *ABCG1* exhibited significant dysregulation in our *in vivo* model, providing experimental evidence for their involvement in this complex pathological environment. Together, these findings suggest that both impaired KATP channel function and disrupted cholesterol transport compromise the brain’s ability to adapt to metabolic stress, thereby increasing susceptibility to neurodegeneration in the context of systemic metabolic dysfunction (Pipatpolkai et al., 2020; Karagiannis et al., 2021; Sancho et al., 2022).

*Caveolin-1 (CAV1)*, identified as a pleiotropic node, connects lipid raft signaling to mitochondrial dynamics, vesicular transport, and synaptic function. Experimental studies show that *CAV1* regulates the balance of mitochondrial fission and fusion by modulating proteins such as DRP1 and mitofusin1, thereby maintaining mitochondrial morphology and function in neurons (Wang et al., 2021; Jiang et al., 2022). Although our findings do not position mitochondrial dysfunction as the primary causal driver, these findings suggest that metabolic stress on lipid and energy pathways—mediated in part by *CAV1*—can accelerate disease progression and create overlapping vulnerabilities across metabolic and neurodegenerative phenotypes (Díaz-Valdivia et al., 2022). The experimental identification of *CAV1* as a key target in our *in vivo* screen provides additional support for its relevance in this interconnected network.

The transcriptomic profiles support these pathway-level interpretations. Our own hippocampal sequencing revealed that the AD-like pathology was characterized by widespread and complex changes in gene expression, particularly in pathways related to lipid metabolism, energy homeostasis, synaptic function, and neuroinflammation, whereas metabolic disorders like T2D and obesity show more focal and specific transcriptomic alterations. High-performing predictive gene sets in AD and VaD often involve genes linked to carbohydrate homeostasis and extracellular protein localization, reflecting the integration of metabolic and neurodegenerative processes at the molecular level. Machine learning models, such as the FT-Transformer, have demonstrated strong discriminatory power for classifying AD and VaD based on distinct expression patterns within lipid and energy pathways, suggesting that these transcriptomic signatures are robust and biologically informative for disease detection (Uzuner Odongo et al., 2025). Such transcriptomic features could be harnessed for early risk detection, particularly in metabolic disorder populations at increased risk of dementia (Guo et al., 2024; Pinheiro et al., 2024; Chaudhuri et al., 2025). However, we acknowledge that the exceptionally high AUCs (>0.94) observed for AD and VaD warrant careful interpretation regarding potential overfitting. However, we posit that these scores reflect the profound biological distinction between the groups: the datasets for neurodegenerative disorders are derived from post-mortem brain tissue exhibiting end-stage pathology (e.g., massive neuronal loss and gliosis), which creates a transcriptomic signal-to-noise ratio far superior to that of early-stage metabolic conditions. Thus, while our rigorous 10-fold cross-validation supports the internal validity of these models, we emphasize that these metrics represent the discrimination of established pathology rather than early clinical diagnosis. Future studies utilizing independent, external cohorts are essential to confirm the generalizability of these signatures.

Our chemical enrichment and docking analyses, mapped against the experimentally prioritized targets, highlight the potential influence of external exposures and pharmacological agents on these shared pathways. Valproic acid, for example, was predicted to bind and potentially activate *SIRT1*, a key regulator of mitochondrial biogenesis and stress response, consistent with prior evidence of neuroprotective and metabolic benefits associated with *SIRT1* activation (Yuan et al., 2016; Xu et al., 2018). In contrast, environmental toxicants like Benzo [a]pyrene (BaP) have been shown to interact with multiple network targets, leading to mitochondrial dysfunction, impaired mitochondrial quality control, and increased oxidative stress, which contribute to neuronal injury and cognitive impairment (Kang et al., 2020; Goveas et al., 2023; Zhao et al., 2023). These findings underscore the importance of gene–environment interactions, as therapeutic activation of protective targets like *SIRT1* or mitigation of toxic exposures such as BaP could modulate disease risk and progression in opposing directions. This highlights the potential for targeted interventions to influence both metabolic and neurodegenerative trajectories by addressing modifiable environmental and pharmacological factors (Kim et al., 2024).

These integrated analyses support a model in which lipid metabolism serves as a primary interface between systemic metabolic status and neuronal health, while energy regulation, including mitochondrial-linked processes, acts as a secondary vulnerability that may amplify pathology under metabolic stress. This conceptual framework aligns with recent longitudinal studies linking midlife T2D and dyslipidemia to later-life cognitive decline (Sebastian et al., 2023; Wee et al., 2023; Choudhary, 2025), as well as experimental findings showing that modulation of lipid transport and mitochondrial resilience can mitigate neurodegenerative phenotypes (Cunnane et al., 2020; Han et al., 2023; Yin, 2023; Munteanu et al., 2024). From a translational perspective, the identified gene targets, including *APOE*, *SORL1*, *LDLR*, *CPT1A*, and *ABCC8*, represent promising points of intervention. Therapeutic strategies aimed at restoring lipid homeostasis and maintaining energy flexibility, alongside environmental risk mitigation, may offer synergistic benefits for slowing or preventing disease progression (Bou Matar et al., 2025). Importantly, we explicitly acknowledge that our multi-modal framework incorporates a sequential dependence, where downstream transcriptomic and machine learning analyses operate within feature spaces pre-defined by upstream genetic findings. While this approach precludes the use of the machine learning models as an independent orthogonal validation of the genetic associations, it serves a distinct and critical purpose: biological prioritization. By constraining the feature space to genetically implicated variants, our analysis functions as a ‘biological sieve’ to determine which susceptibility genes remain transcriptionally active and diagnostically predictive within the established pathological tissue environment. The metabolic and energy pathways identified herein therefore represent a refined intersection of germline potential and somatic reality, confirming that these genetic risk factors are not merely latent liabilities but are mechanistically engaged in the active disease state. Furthermore, the identification of specific targets such as *CPT1A* (mitochondrial fatty acid oxidation) and *ABCC8* (energy sensing)—beyond the canonical *APOE* signal—demonstrates the study’s biological significance. discriminating established pathology It pinpoints actionable metabolic vulnerabilities linking systemic disease to neurodegeneration (Tovar et al., 2013; Liang, 2023), thereby moving beyond generic pathway associations.

Several limitations should be acknowledged. First, while our *in vivo* model provides crucial transcriptomic evidence for prioritizing genetically-implicated candidates, the current study does not include functional perturbation experiments (e.g., gene knockdown or overexpression) to establish definitive causal mechanisms for the prioritized genes. The primary function of our murine model is to validate that genetically-implicated candidates, such as *APOE, CLU,* and *LDLR*, exhibit actual transcriptional dysregulation in a pathologically relevant neurodegenerative context. This transcriptomic validation serves as a critical filter, distinguishing genes with demonstrated *in vivo* pathological relevance from those identified solely through statistical associations in human GWAS data. However, establishing the precise functional roles of these genes requires additional mechanistic studies. Specifically, future work should use: (1) CRISPR/Cas9-mediated gene editing in primary neuronal cultures and iPSC-derived neurons to assess the impact of candidate gene disruption on lipid metabolism, synaptic function, and amyloid pathology; (2) AAV-mediated overexpression or knockdown *in vivo* to evaluate gene-specific contributions to cognitive decline and neuropathology in metabolic disease contexts; and (3) cell-type-specific interventions using conditional knockout models to dissect the contributions of neuronal *versus* glial compartments. By explicitly delineating the current scope of our experimental validation, we provide a transparent foundation for the next phase of mechanistic inquiry. We also recognize that the D-galactose/sodium nitrite mouse model used for validation represents a chemical mimic of aging and oxidative stress rather than a transgenic model of amyloid proteinopathy. While this model does not develop the classic amyloid plaques or neurofibrillary tangles characteristic of human AD, it robustly recapitulates the synaptic loss, neuroinflammation, and metabolic/lipid peroxidation characteristic of sporadic, age-related neurodegeneration (Shwe et al., 2018). Given our study’s focus on the metabolic-neurocognitive interface—specifically lipid dysregulation and environmental stress responses—this model provides a biologically relevant context for validating transcriptional susceptibility to systemic metabolic injury.

Second, MR methods, while powerful for causal inference, are sensitive to horizontal pleiotropy and instrument heterogeneity, especially for traits with shared polygenic architectures such as lipid metabolism and neurodegeneration. Although sensitivity analyses can mitigate these concerns, residual bias cannot be fully excluded. Besides, we explicitly recognize the limitations associated with using parental history of AD as a proxy phenotype. While this approach enhances statistical power, it is susceptible to survivor bias. The observed “protective” effect of parental dementia on NAFLD may be partially attributable to a selection artifact, where individuals with severe metabolic dysfunction succumb to cardiovascular mortality before reaching the age of dementia onset (Mayeda et al., 2016). Second, our inclusion of the APOE locus introduces horizontal pleiotropy. However, rather than viewing these signals solely as statistical artifacts, we interpret them as evidence of “biological pleiotropy,” where a single genetic hub drives simultaneous divergence in metabolic and neurocognitive pathways (Andrews et al., 2021). Our use of Steiger filtering confirms the directionality of these associations, suggesting they reflect a genuine, genetically anchored interface. Third, transcriptomic datasets used here are bulk tissue profiles, limiting resolution for cell-type-specific changes, which is particularly relevant given the cellular diversity of the brain and metabolic tissues. Our *in vivo* transcriptomics were also based on bulk hippocampal tissue, and future work integrating single-cell and spatial transcriptomics will help localize lipid and energy pathway disruptions to specific cell populations, clarifying mechanisms at a finer resolution. Fourth, we explicitly acknowledge that the molecular docking and CTD network analyses presented herein represent computational predictions of binding affinity and database-derived associations, respectively. They do not constitute *in vivo* or *in vitro* validation of physical target engagement. While these *in silico* models provide a rationale for exploring specific chemical-gene interactions (e.g., Valproic acid-SIRT1), definitive confirmation requires future biophysical assays (e.g., Surface Plasmon Resonance or Cellular Thermal Shift Assays) to verify direct binding kinetics and functional occupancy (Pinzi and Rastelli, 2019). Finally, the GWAS datasets leveraged for MR were predominantly of European ancestry, limiting generalizability across diverse populations. Future studies should expand to multi-ancestry cohorts and longitudinal designs, incorporating multi-omics data to better capture population-specific risk architecture and temporal dynamics.

## Conclusion

This study elucidates the causal architectures linking metabolic and neurocognitive disorders. Through network-informed Mendelian randomization, we first identified six functional modules with complex bidirectional and context-dependent associations, such as T2D elevating dementia risk while obesity showed protective effects against parental AD history. Genetic dissection uncovered asymmetric drivers, including recurrent loci like *APOE/TOMM440* and *FTO/BDNF*. To anchor these genetic signals in a biological context, we performed transcriptomic analysis on an *in vivo* AD-like model. This pivotal step identified a core network of 13 dysregulated genes, including the specialist hub *SORL1* and generalist nodes *APOE* and *CAV1*. The dysregulation of this signature provides direct experimental support for lipid dysregulation as a central shared mechanism. Finally, chemical enrichment and molecular docking revealed that pleiotropic agents, including valproic acid and benzo [a]pyrene, converge on these validated nodes. In sum, this integrated analysis provides a multi-layered line of evidence for shared molecular vulnerabilities and offers a set of experimentally-prioritized targets as a foundation for developing diagnostic models and therapeutic interventions aimed at lipid homeostasis to address these interconnected pathologies. While functional perturbation experiments are needed to definitively establish causal mechanisms, our transcriptomic validation in a pathologically relevant *in vivo* model, combined with converging evidence from human genetics and computational chemistry, provides a robust platform for hypothesis-driven mechanistic studies and translational development.

## Data Availability

Publicly available datasets were analyzed in this study. This data can be found here: Genetic association summary statistics were obtained from IEU OpenGWAS (https://gwas.mrcieu.ac.uk/; https://mrcieu.github.io/software/opengwas/), the NHGRI-EBI GWAS Catalog (https://www.ebi.ac.uk/gwas/), and the FinnGen Consortium (https://www.finngen.fi/). Transcriptomic datasets were downloaded from the Gene Expression Omnibus (GEO) (https://www.ncbi.nlm.nih.gov/geo/). Curated chemical—gene interactions were retrieved from the Comparative Toxicogenomics Database (CTD) (https://ctdbase.org/), and protein structures from the Protein Data Bank (PDB) (https://www.rcsb.org/). Ligand structures were obtained from PubChem (https://pubchem.ncbi.nlm.nih.gov/).
