# Supplementary material for: Integrating causal human genetics and In vivo transcriptomics to uncover a shared lipid-centric architecture in metabolic and neurocognitive disease
Source: Front Mol Biosci. 2026 Jan 27;12:1712198. doi: 10.3389/fmolb.2025.1712198 (PMC12887701; doi:10.3389/fmolb.2025.1712198)
Supplement: Supplementary file 1 [file Supplementaryfile1.docx]

**Supplementary Figures**

[Supplementary Figure 1. Comprehensive Performance Evaluation and Statistical Comparison of Machine Learning Models for Neurodegenerative Disease Classification. 2](#_Toc205394688)

[Supplementary Figure 2. Comparative Performance Analysis and Statistical Evaluation of Machine Learning Models for Metabolic Disease Classification. 4](#_Toc205394689)

[Supplementary Figure 3. Performance of the Top 10 Predictive Gene Sets for Each Disease Cohort. 6](#_Toc205394690)

[Supplementary Figure 4. Differential Gene Expression Dashboards Revealing Distinct Transcriptional Architectures Across Disease Cohorts. 8](#_Toc205394691)


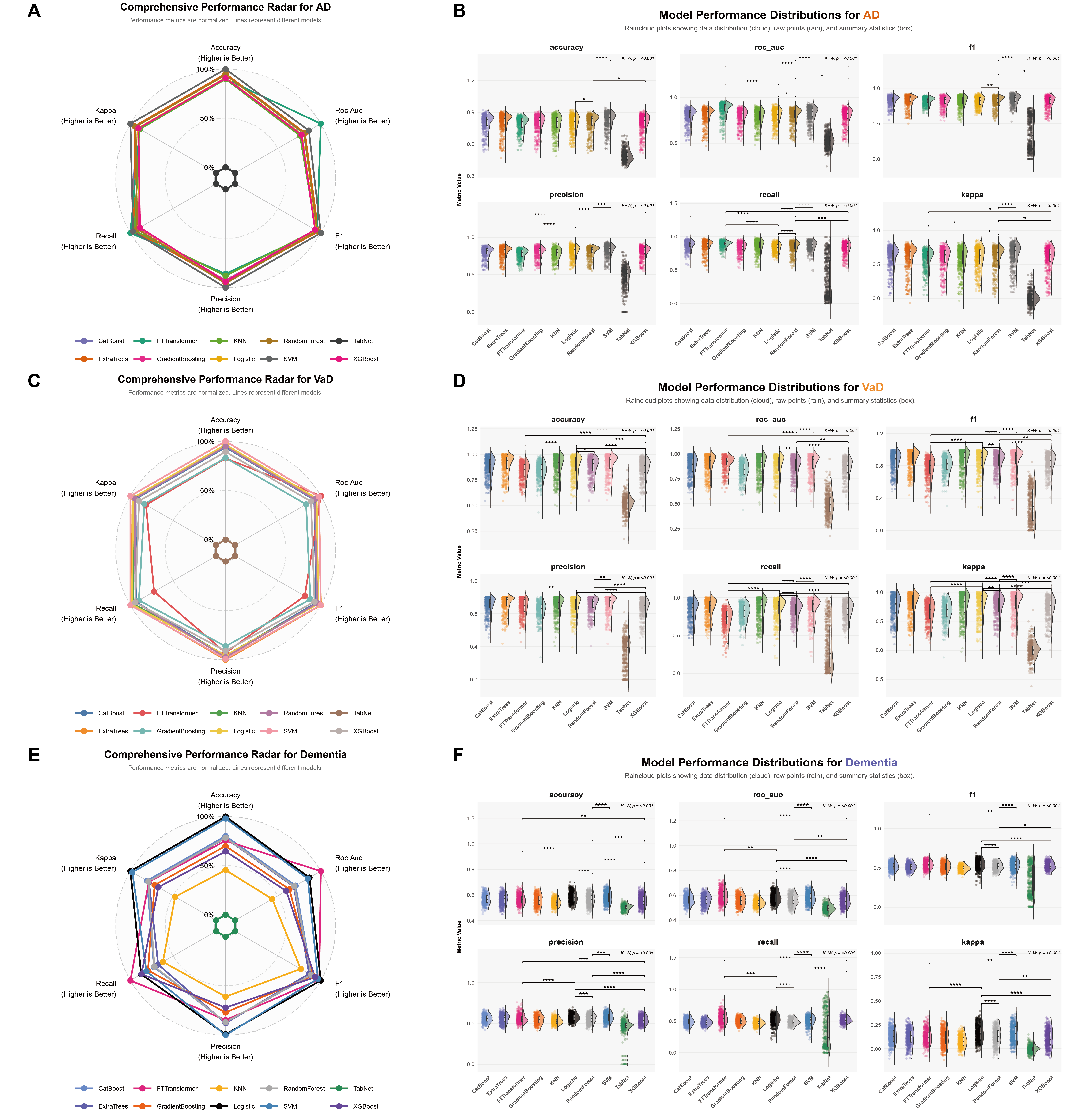


# Supplementary Figure 1. Comprehensive Performance Evaluation and Statistical Comparison of Machine Learning Models for Neurodegenerative Disease Classification.

This figure provides a detailed evaluation of ten machine learning models for classifying Alzheimer's Disease (AD), Vascular Dementia (VaD), and general Dementia based on transcriptomic signatures, demonstrating strong to excellent predictive performance.

- **(A) Comprehensive Performance Radar for AD.** This plot provides a multi-metric overview of each model's normalized performance. The Support Vector Machine (SVM) demonstrates the most robust and balanced profile, achieving high scores across multiple metrics. In contrast, the FT-Transformer shows a specialized strength, excelling in ROC AUC but showing lower performance on other metrics. The TabNet model's profile is confined to the center, indicating a failure to learn meaningful patterns.
- **(B) Model Performance Distributions for AD.** These raincloud plots show performance distributions and include pairwise statistical comparisons (Wilcoxon rank-sum test). The SVM (mean AUC = 0.825, accuracy = 0.828) significantly outperforms most models across balanced metrics like accuracy and AUC (*P* < 0.0001 vs. RandomForest). The FT-Transformer's specialized strength is statistically confirmed, achieving a significantly higher AUC (mean = 0.874) and recall than all other models (*P* < 0.0001).
- **(C) Comprehensive Performance Radar for VaD.** In this high-signal cohort, multiple models show excellent performance. ExtraTrees and SVM display the most balanced, high-performing profiles, extending to the outer edges of the radar.
- **(D) Model Performance Distributions for VaD.** The statistical analysis confirms that ExtraTrees (mean AUC = 0.899, accuracy = 0.903) and SVM (mean AUC = 0.900, accuracy = 0.904) are top-tier performers, significantly outperforming most others in F1-score and kappa (*P* < 0.0001). The FT-Transformer's AUC (mean = 0.908) is significantly higher than that of other strong models like XGBoost (*P* < 0.0001).
- **(E) Comprehensive Performance Radar for Dementia.** For this lower-signal cohort, overall performance is more modest. SVM and Logistic Regression emerge as the most effective models, showing the most balanced performance profiles.
- **(F) Model Performance Distributions for Dementia.** Statistical comparisons confirm that SVM (mean AUC = 0.579) and Logistic Regression (mean AUC = 0.581) significantly outperform other models in balanced metrics like kappa (*P* < 0.0001), while the FT-Transformer once again excels in AUC (mean = 0.594), highlighting its ability to discriminate classes even in challenging contexts.


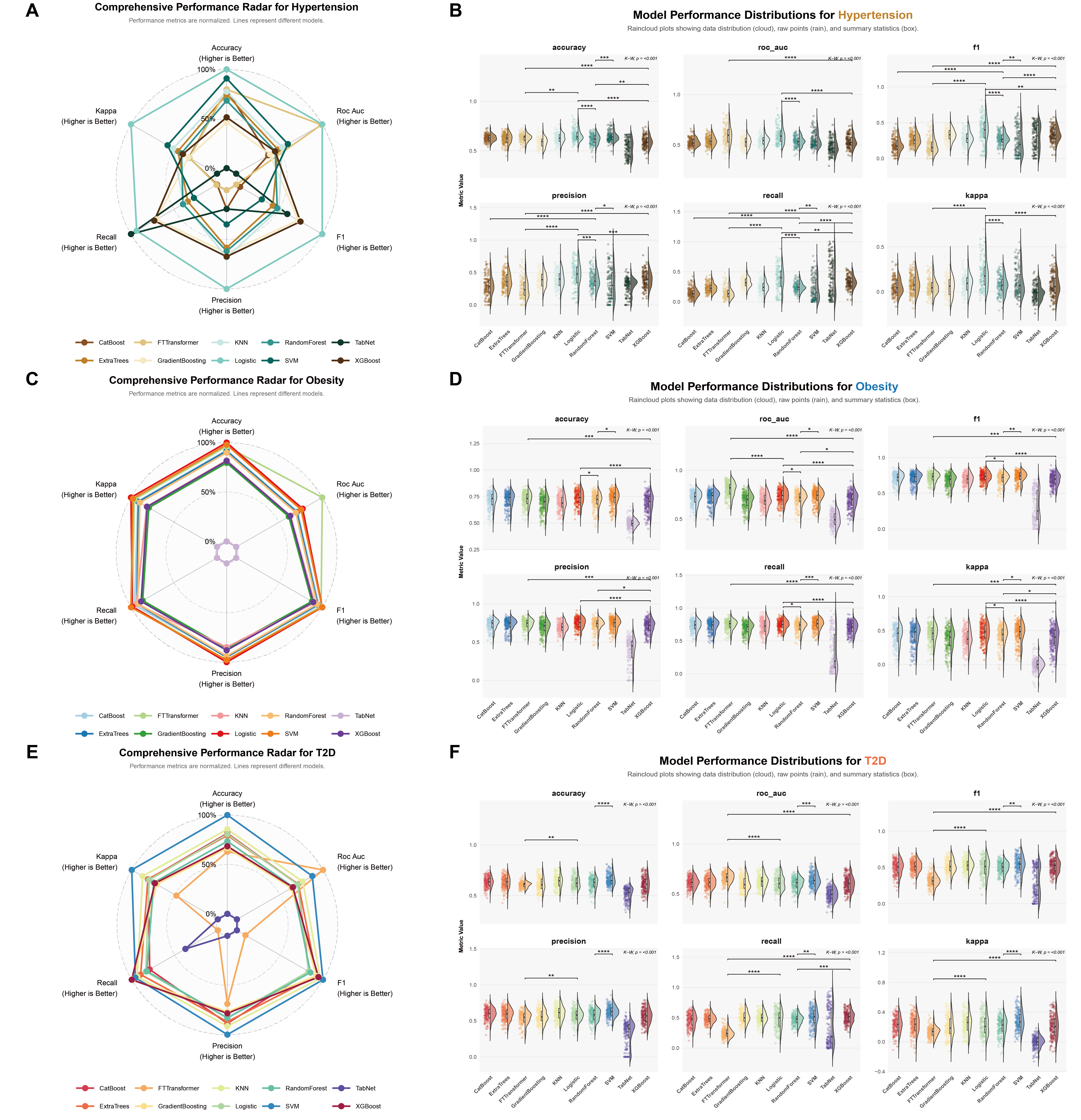


# Supplementary Figure 2. Comparative Performance Analysis and Statistical Evaluation of Machine Learning Models for Metabolic Disease Classification.

This figure presents a detailed performance evaluation of ten machine learning models for classifying Hypertension, Obesity, and Type 2 Diabetes (T2D) using transcriptomic signatures, revealing more modest and variable predictive power compared to Neurocognitive Disorder.

- **(A) Comprehensive Performance Radar for Hypertension.** This plot illustrates the limited predictive power of all models for this challenging cohort, with most profiles clustered near the center. The simpler Logistic Regression model shows the most balanced, albeit modest, performance.
- **(B) Model Performance Distributions for Hypertension.** Statistical analysis confirms that Logistic Regression (mean AUC = 0.598, accuracy = 0.658) holds a slight but significant advantage over most other models in overall accuracy and kappa (e.g., *P* < 0.0001 vs. RandomForest). In this low-signal context, the FT-Transformer shows a trade-off, with significantly lower F1-scores and recall than Logistic Regression (*P* < 0.0001 for both).
- **(C) Comprehensive Performance Radar for Obesity.** For this cohort, the FT-Transformer and Logistic Regression emerge as the top-performing models, with their profiles extending furthest on the ROC AUC and other axes, indicating superior performance.
- **(D) Model Performance Distributions for Obesity.** The raincloud plots reveal a performance trade-off: the FT-Transformer achieves a significantly higher AUC (mean = 0.804) than other models (*P* < 0.0001 vs. Logistic Regression), while Logistic Regression (mean AUC = 0.735) performs significantly better in balanced metrics like accuracy (*P* = 0.033 vs. RandomForest) and kappa (P = 0.034).
- **(E) Comprehensive Performance Radar for T2D.** The Support Vector Machine (SVM) demonstrates the most consistently robust performance, as shown by its relatively large and balanced shape on the radar.
- **(F) Model Performance Distributions for T2D.** Statistical comparisons confirm that SVM (mean AUC = 0.642, accuracy = 0.660) is the most consistently superior model, significantly outperforming RandomForest in accuracy, precision, and kappa (*P* < 0.0001). The FT-Transformer once again demonstrates a significant advantage in AUC but shows weaker performance in balanced metrics like the F1-score compared to SVM and Logistic Regression.


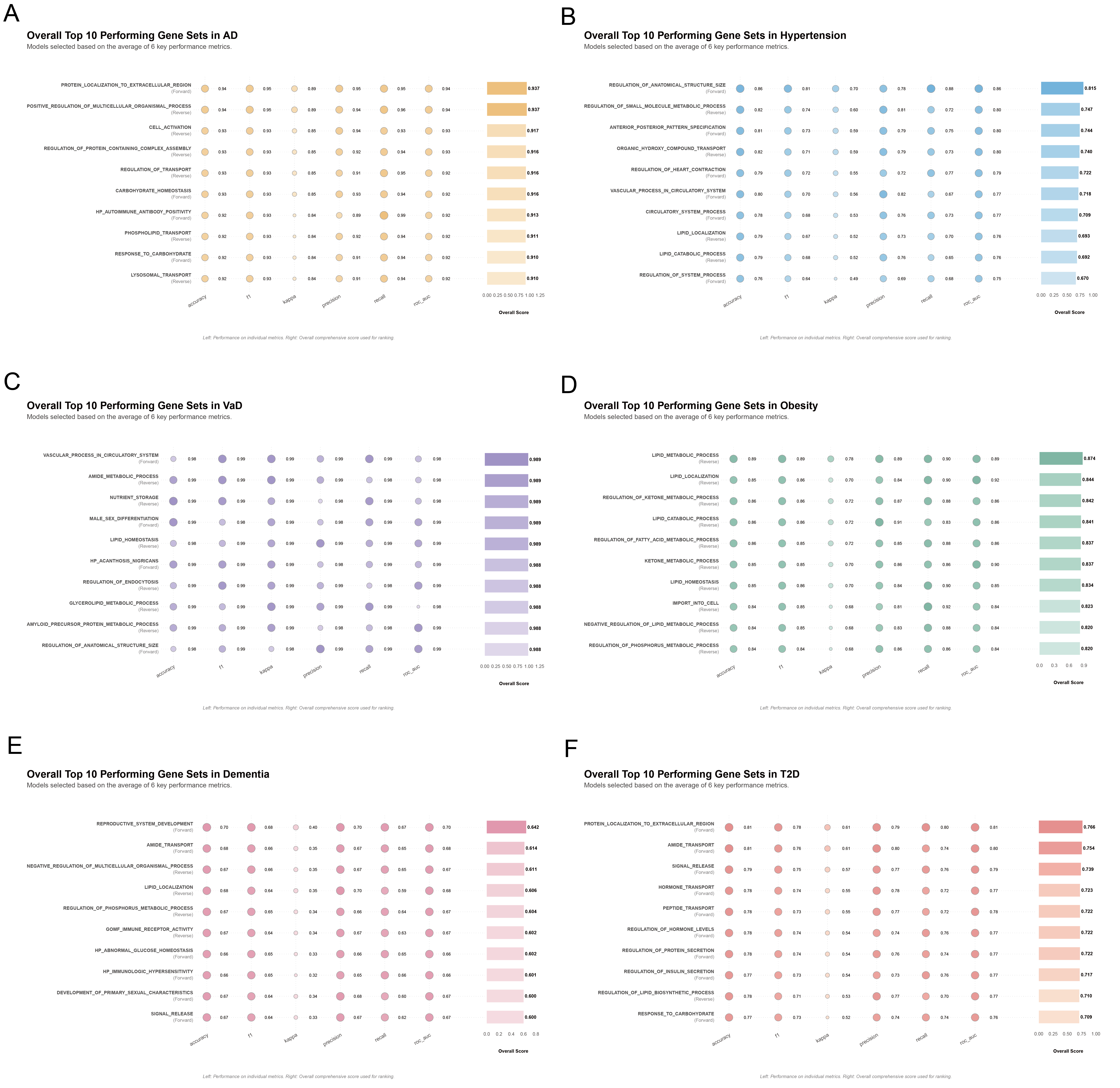


# Supplementary Figure 3. Performance of the Top 10 Predictive Gene Sets for Each Disease Cohort.

This figure displays the top 10 performing gene sets (pathways) for each of the six disease cohorts, ranked by an overall score derived from six key performance metrics. For each pathway, the left panel shows its performance on individual metrics (Accuracy, F1-Score, Kappa, Precision, Recall, ROC AUC), while the right panel displays its ranked overall score. The analysis reveals that the diagnostic potential of transcriptomic signatures varies significantly across diseases, with neurodegenerative cohorts showing much stronger predictive signals.

- **(A) Alzheimer's Disease (AD):** Predictive performance was high, with the top gene set, *PROTEIN LOCALIZATION TO EXTRACELLULAR REGION*, achieving an overall score of 0.937 (AUC = 0.943, Accuracy = 0.944). Other highly predictive pathways included *POSITIVE REGULATION OF MULTICELLULAR ORGANISMAL PROCESS* and *CELL ACTIVATION*, highlighting the diagnostic importance of extracellular protein dynamics and immune regulation.
- **(B) Hypertension:** Predictive signals were modest. The top-performing gene set was *REGULATION OF ANATOMICAL STRUCTURE SIZE*, achieving an overall score of 0.827 (AUC = 0.863, Accuracy = 0.856).
- **(C) Vascular Dementia (VaD):** Gene sets demonstrated exceptional predictive power, with the top 10 pathways all achieving overall scores above 0.98. Key examples include *VASCULAR PROCESS IN CIRCULATORY SYSTEM* (Overall Score = 0.989), *AMYLOID PRECURSOR PROTEIN METABOLIC PROCESS*, and *CARBOHYDRATE HOMEOSTASIS*, underscoring the strong transcriptomic signatures related to vascular function, amyloid processing, and glucose regulation.
- **(D) Obesity:** Performance was moderate, with pathways primarily linked to lipid biology. *LIPID METABOLIC PROCESS* was the top performer with an overall score of 0.874 (AUC = 0.890, Accuracy = 0.890), followed by *LIPID LOCALIZATION* (Overall Score = 0.874).
- **(E) Dementia:** For the general dementia cohort, predictive signals were weakest. The top-ranked gene set was *REPRODUCTIVE SYSTEM DEVELOPMENT*, achieving a modest overall score of 0.674 (AUC = 0.698).
- **(F) Type 2 Diabetes (T2D):** The top-ranked gene set was *PROTEIN LOCALIZATION TO EXTRACELLULAR REGION*, which yielded an overall score of 0.788 (AUC = 0.807, Accuracy = 0.808).


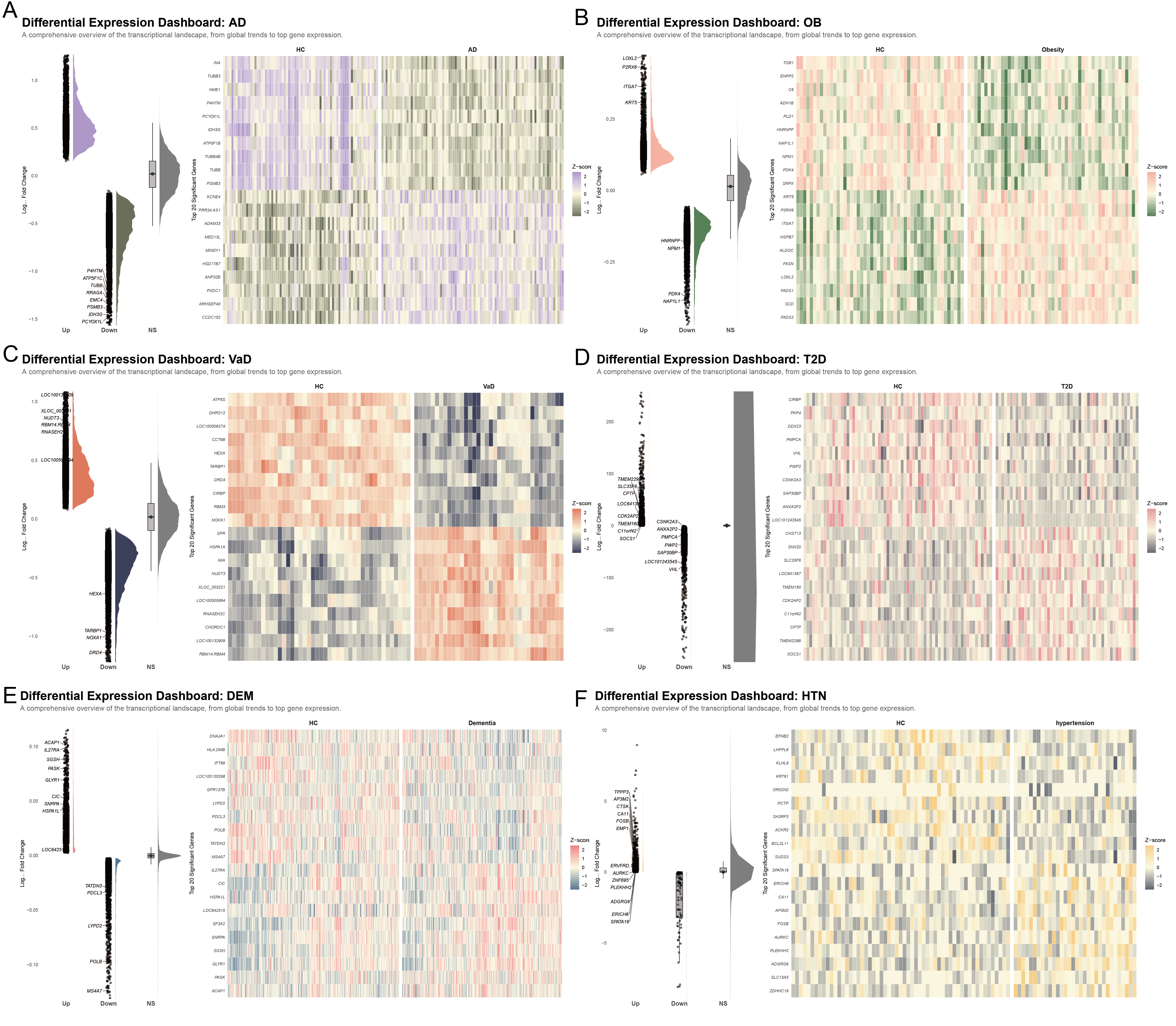


# **Supplementary Figure 4. Differential Gene Expression Dashboards Revealing Distinct Transcriptional Architectures Across Disease Cohorts.**

This figure presents a comprehensive overview of the transcriptional landscape for each of the six disease cohorts. Each dashboard consists of two parts: a left panel showing the distribution of log-fold changes (logFC) for all significantly up-regulated, down-regulated, and non-significant genes, and a right panel displaying a heatmap of the Z-score normalized expression for the top 20 most significant genes across healthy controls (HC) and disease samples. The analysis highlights two distinct patterns of dysregulation.

- **Neurodegenerative disorders (A, C, E)** are characterized by widespread but modest transcriptional perturbations. **(C) Vascular Dementia (VaD)** and **(A) Alzheimer's Disease (AD)** show the most extensive changes, with thousands of differentially expressed genes (VaD: 5,918 down, 5,477 up; AD: 4,850 down, 3,305 up), though the average magnitude of change is modest (mean absolute logFC ≈ 0.41–0.61). This is visually represented by the broad distributions in the logFC plots and clear but not extreme expression patterns in the heatmaps. **(E) Dementia (DEM)** shows a similar but more subtle profile, with the lowest average change magnitude (≈ 0.03).
- **Metabolic disorders (B, D, F)** are typified by more focal yet pronounced gene dysregulation. **(D) Type 2 Diabetes (T2D)** exhibits the most extreme alterations, with an exceptionally high average absolute logFC of 52.80 for its 1,073 down-regulated genes, as seen in the highly skewed logFC plot. **(F) Hypertension (HTN)** has the most circumscribed signature with the fewest affected genes, but the changes are substantial (avg. abs. logFC = 3.11 for down-regulated genes). **(B) Obesity (OB)** presents a hybrid profile with a large number of dysregulated genes but with a smaller magnitude of change (avg. abs. logFC ≈ 0.16) compared to other metabolic disorders.
